# The Role of Mitotic Kinases and the RZZ Complex in Kinetochore-Microtubule Attachments: Doing the Right Link

**DOI:** 10.3389/fcell.2022.787294

**Published:** 2022-01-28

**Authors:** João Barbosa, Claudio E. Sunkel, Carlos Conde

**Affiliations:** ^1^ i3S, Instituto de Investigação e Inovação em Saúde da Universidade do Porto, Porto, Portugal; ^2^ IBMC - Instituto de Biologia Molecular e Celular, Universidade do Porto, Porto, Portugal; ^3^ ICBAS - Instituto de Ciências Biomédicas Abel Salazar, Universidade do Porto, Porto, Portugal

**Keywords:** kinetochore-microtubule attachments, kinetochore, POLO/PLK1, AURORA B, RZZ complex, mitosis

## Abstract

During mitosis, the interaction of kinetochores (KTs) with microtubules (MTs) drives chromosome congression to the spindle equator and supports the segregation of sister chromatids. Faithful genome partition critically relies on the ability of chromosomes to establish and maintain proper amphitelic end-on attachments, a configuration in which sister KTs are connected to robust MT fibers emanating from opposite spindle poles. Because the capture of spindle MTs by KTs is error prone, cells use mechanisms that sense and correct inaccurate KT-MT interactions before committing to segregate sister chromatids in anaphase. If left unresolved, these errors can result in the unequal distribution of chromosomes and lead to aneuploidy, a hallmark of cancer. In this review, we provide an overview of the molecular strategies that monitor the formation and fine-tuning of KT-MT attachments. We describe the complex network of proteins that operates at the KT-MT interface and discuss how AURORA B and PLK1 coordinate several concurrent events so that the stability of KT-MT attachments is precisely modulated throughout mitotic progression. We also outline updated knowledge on how the RZZ complex is regulated to ensure the formation of end-on attachments and the fidelity of mitosis.

## Introduction

**FIGURE 1:**
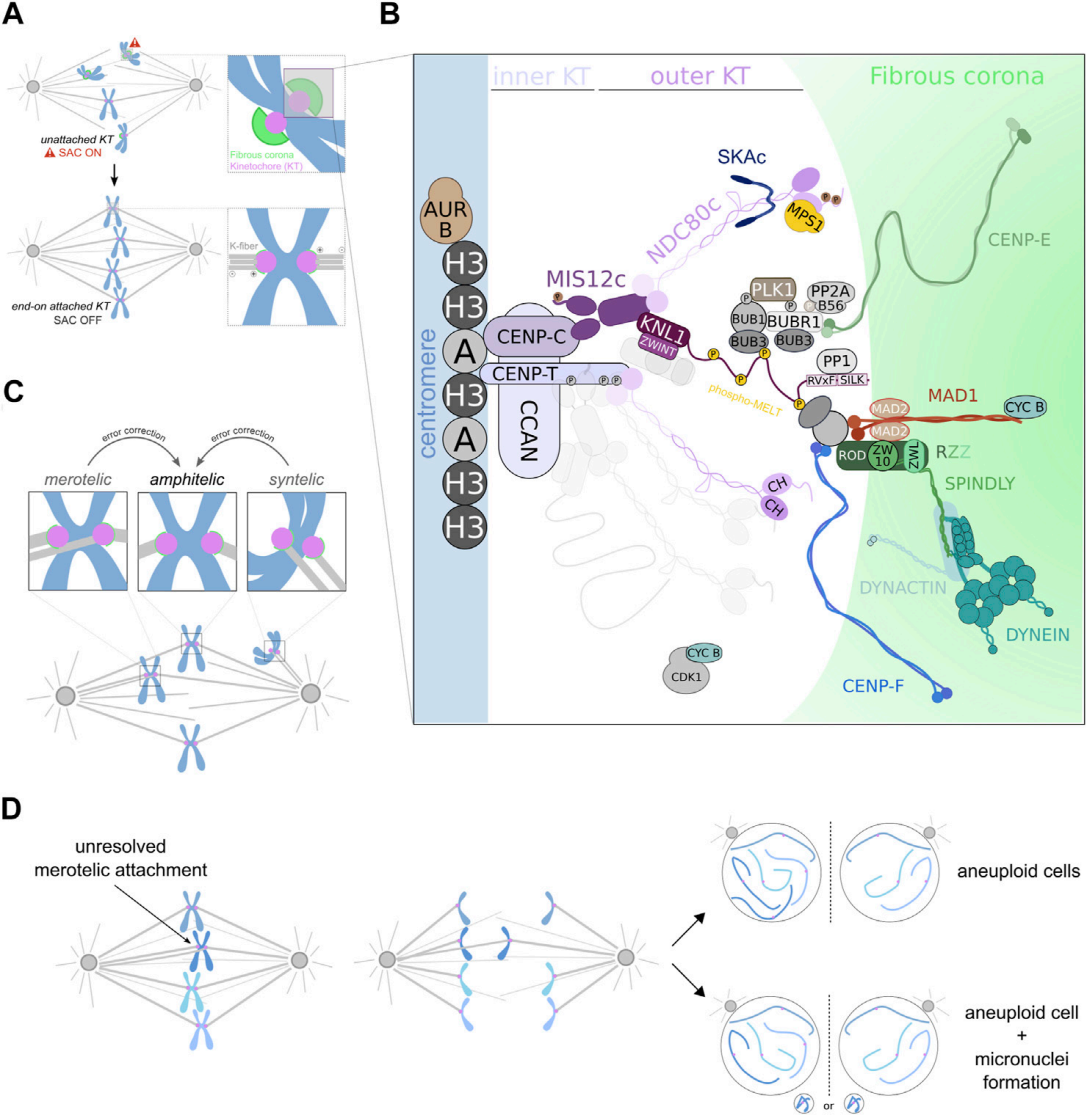
Establishment of appropriate KT-MT attachments supports faithful chromosome segregation. **(A)** During mitosis, chromosomes interact with spindle MTs (represented in gray) through KTs (represented in magenta), a proteinaceous structure that assembles on centromeres. The outermost region of the KT, known as the fibrous corona (represented in green), dynamically expands and compacts during prometaphase to facilitate chromosome capture by spindle MTs. KTs that lack MT attachment generate a SAC response that results in the formation of a diffusible effector complex that ultimately prevents premature chromosome segregation. Once all chromosomes are properly attached to spindle MTs, the SAC is silenced and chromosome segregation ensues. **(B)** Simplified view of the KT composition at centromeres. The CCAN, including CENP-C and CENP-T, localizes closer to centromeric CENP-A (A) nucleosomes, which are intercalated between canonical H3 nucleosomes. In turn, the KMN network, which encompasses Spc105/KNL1:ZWINT, the MIS12c and the NDC80c, faces the opposite direction toward the MT binding region. In yeast and vertebrates, both CENP-C and CENP-T can recruit KMN components. This requires the activity of AURORA B and CDK1:CYCB. Importantly, AURORA B can also phosphorylate the N-terminal tail domain in NDC80/HEC1 and inhibit its MT binding activity. In contrast, the MT-binding capacity of the KT can be strengthened by NDC80-dependent recruitment of SKAc. The NDC80c also constitutes a binding site for MPS1, which phosphorylates several MELT motifs (phospho-MELT) in KNL1, triggering the recruitment of a number of SAC components to KTs. KNL1 also contains PP1-binding motifs close to its N-terminus, whose recruitment is important for SAC silencing. Additional proteins, namely the RZZc and SPINDLY, are recruited to KTs and catalyze the formation of the fibrous corona during prometaphase. This structure also contains DYNEIN:DYNACTIN, CENP-E and CENP-F, all of which facilitate initial interactions between KTs and MTs. **(C)** Schematic representation of different KT-MT attachment configurations that can be established during the course of mitosis. Both syntelic and merotelic interactions must be converted into amphitelic attachments to avoid chromosome mis-segregation. **(D)** The presence of unresolved merotelic attachments often leads to the occurrence of lagging chromosomes during anaphase which can preclude their correct segregation. A lagging chromosome can eventually segregate to the wrong daughter cell, giving rise to aneuploid progeny. Alternatively, it can move toward the right chromosome mass, but form a micronucleus as a result of delayed segregation. Micronuclei accumulate DNA damage that can cause subsequent chromosomal rearrangements and, hence, genomic instability.

The distribution of a full complement of chromosomes to each daughter cell at the end of mitosis represents a decisive step for the maintenance of genomic stability. Chromosome segregation relies on microtubule (MT)-dependent transport and, therefore, the establishment of appropriate MT interactions with centromere-localized kinetochores (KTs) constitutes a pre-requisite for faithful genome partition (Figure 1A). Importantly, these interactions also contribute to the correct positioning (congression) of chromosomes at the equatorial plane of the spindle during early mitosis, ideally with sister KTs binding MTs extending from opposite spindle poles (amphitelic attachments) (Figure 1A). In vertebrates, over 100 different proteins have been identified at KTs, providing these structures the capacity to perform different functions required for mitotic fidelity (Figure 1B) (Joglekar and Kukreja, 2017). For instance, a subset of KT proteins assembles a molecular machinery capable of supporting MT binding and transducing the force generated by MT dynamics at the KT-MT interface to power chromosome movement (Maiato et al., 2017). Not surprisingly, KTs also dock proteins that promote the establishment of proper KT-MT attachments. Given that erroneous attachment configurations can arise during mitosis, the activity of error correction mechanisms critically favors the formation of appropriate interactions between KTs and MTs (discussed below). Incorrect interactions are typically identified as syntelic—where both sister KTs bind MT growing from the same spindle pole—or merotelic—where a single KT attaches to MTs emanating from opposite spindle poles (Figure 1C). Both attachment configurations must be resolved into proper bioriented amphitelic interactions (Figure 1C) (Lampson and Grishchuk, 2017). Failure to correct erroneous attachments can lead to chromosome mis-segregation and aneuploidy (Figure 1D). This creates a genetic imbalance that is often associated with developmental defects and malignant tumors (Schukken and Foijer, 2018). Importantly, KTs also accumulate Spindle Assembly Checkpoint (SAC) proteins that signal the lack of MT attachment and prevents cells from prematurely exiting mitosis with unattached chromatids (Figure 1A) (Joglekar, 2016). All these regulatory mechanisms involve common elements that facilitate the coordination of the complex set of biochemical activities that occur within KTs. In this context, the KMN network, a protein complex that localizes at the outer KT region, emerges as a key player by integrating KT-MT attachment- and SAC-related functions (Joglekar, 2016). The presence of kinases and phosphatases at KTs and centromeres ensures the fine regulation of all of these mitotic events (Saurin, 2018; Moura and Conde, 2019).

The KMN network refers to a macromolecular assembly of 3 protein sub-complexes—Spc105/KNL1, MIS12 and NDC80/HEC1—where each component is associated with different functions (Figure 1B) (Cheeseman et al., 2004; Cheeseman et al., 2006). While the MIS12 complex (MIS12c) connects the KMN network to the chromatin-facing inner KT structure, KNL1 acts as a scaffold for most SAC proteins to localize. The NDC80 complex (NDC80c) has MT-binding activity and establishes the KMN as the primary interface for KT-MT attachments (Cheeseman et al., 2006). Importantly, the KMN network also supports, directly or indirectly, the recruitment of numerous other KT proteins that function in parallel with it in the formation of KT-MT interactions (Figure 1B). Some of these proteins are able to bind to MTs (being broadly referred as microtubule-associated proteins or MAPs) and include the motor proteins DYNEIN and CENP-E, as well as auxiliary proteins such as CENP-F, the SKA complex (SKAc), ASTRIN:SKAP, CLASP 1/2, CDT1 or chTOG (Maiato et al., 2017; Monda and Cheeseman, 2018). However, the requirement for a particular MAP may depend on whether the prevailing KT-MT interaction interface is established between KTs and either the MT lattice—lateral interactions—or the plus ends of MTs—end-on interactions. Nonetheless, accurate chromosome segregation relies on the conversion of lateral to end-on interactions which, in turn, are able to couple the force generated by MT dynamics to power chromosome movement and segregation (Maiato et al., 2017). The combined activities of the KMN network and auxiliary proteins directly contribute to the overall capacity of the KT to withstand forces exerted by spindle MTs. EM-based studies of the KT-MT interface in cells from different species allowed the quantification of the average number of MTs that are embedded in the KT (end-on attached) of an aligned chromosome. These MT bundles, collectively referred as a KT-fiber, contain ∼17–20 MTs in human cells (Wendell et al., 1993; Dudka et al., 2018), ∼11 MTs in *Drosophila* S2 cells (Maiato et al., 2006), ∼25 MTs per μm (Maiato et al., 2017) of KT surface (holocentric chromosomes) in *C. elegans* one-cell embryos, ∼2–3 MTs in *S. pombe* cells and 1 MT in *S. cerevisiae* (McIntosh et al., 2013). Successfully building a robust KT-MT interface helps to endure forces resulting from MT depolymerization that ultimately drive chromosome segregation (Auckland and McAinsh, 2015), thus likely contributing to decrease the incidence of lagging chromosomes during anaphase (Dudka et al., 2018). Nevertheless, the formation of stable KT-MT end-on attachments is not an irreversible process. The reversible nature of KT-MT interactions is essential for the correction of inappropriately oriented attachments—syntelic and merotelic—and, hence, safeguards genomic stability.

In this review, we outline the complex network of mechanisms controlling dynamic KT-MT interactions, with emphasis on how key mitotic kinases AURORA B and PLK1 regulate their substrates at the KT-MT interface. We also discuss recent advances on our understanding of how the RZZ complex (RZZc) fine-tunes KT-MT attachment stability to ensure the fidelity of chromosome segregation.

## Regulation of KT-MT Interactions

Erroneous KT-MT attachments can result in chromosome gain or loss at the end of mitosis (Figure 1D) (Godek et al., 2015). Before anaphase onset, the SAC monitors the attachment state of chromosomes and delays mitotic progression until all KTs are attached to spindle MTs. However, the SAC fails to directly discriminate between proper and erroneous KT-MT attachments (Khodjakov and Pines, 2010). The existence of error correction mechanisms is therefore critical to ensure that KTs bind to MTs in an appropriate manner. This is particularly important considering that improper attachments may often occur during early mitosis as a result of the stochastic nature of the interactions between KTs and MTs (Figure 1C) (Lampson and Grishchuk, 2017). Notably, syntelic attachments are short-lived and lead to SAC activation in response to increased destabilization of attached MTs, whilst merotelic attachments can escape SAC surveillance. This poses a potential problem given that merotely at anaphase onset can engage the KT in a tug-of-war, where pulling forces from MTs derived from opposite spindle poles cause the chromatid to lag behind (Figure 1D) (Holland and Cleveland, 2012). Interestingly, most lagging KTs eventually segregate to the correct spindle pole (Cimini et al., 2004; Thompson and Compton, 2011). However, the presence of additional mitotic defects (ex, multipolar spindles) can increase the prevalence of merotelic KTs and in such scenarios, the correction mechanisms may not be sufficiently robust to cope with a higher rate of inappropriate KT-MT attachment formation. This can enhance mis-segregation of merotelic KTs, thus leading to the generation of aneuploid cells (Figure 1D) (Thompson and Compton, 2008; Thompson and Compton, 2011; Holland and Cleveland, 2012).

Computational modeling combined with experimental observations defined how basic mechanisms contribute to the formation of amphitelic attachments during mitosis (Lončarek, 2007; Bakhoum et al., 2009a; Paul et al., 2009; Zaytsev and Grishchuk, 2015). First, the conspicuous back-to-back arrangement of sister KTs in aligned chromosomes favors biorientation, as it introduces geometric limitations that bias KTs to face opposite directions. Consequently, sister KTs are more likely to bind to MTs from opposite spindle poles (Lončarek, 2007; Zaytsev and Grishchuk, 2015). This can explain why chromosomes bearing a merotelic KT still segregate to the correct spindle pole. The merotelic KT will build a more robust K-fiber towards the proximal, correct spindle pole than to the distal, less exposed pole. This difference in the robustness of MT bundles creates a force differential that favors the segregation of the chromatid to the correct pole. Moreover, the establishment of correctly bioriented attachments is further promoted by the rotation of the inter-KT axis following a first end-on interaction, which maximizes the beneficial impact of a back-to-back geometry of sister KTs (Paul et al., 2009; Magidson et al., 2015). Nevertheless, the chance to form erroneous interactions is not completely eliminated by these geometric constraints (Magidson et al., 2015; Zaytsev and Grishchuk, 2015). At mitotic entry, chromosomes may assume unfavorable positions that primarily exposes both KTs to one spindle pole (as for syntelic interactions) or a single KT to MTs from both spindle poles (as for merotelic interactions) (Figure 1C). Therefore, additional mechanisms are required to ensure that any mal-oriented KT-MT attachment is resolved and only proper interactions are established and preserved.

The indiscriminate turnover of KT-attached MTs constitutes an essential mechanism to facilitate the correction of erroneous KT-MT attachments (Godek et al., 2015; Zaytsev and Grishchuk, 2015). As chromosomes congress and sister KTs become favorably oriented towards opposite spindle poles, the continuous attachment and detachment of MTs at KTs allows for the selective replacement of improper MTs acquired earlier with correct ones. In mammalian cells, MT turnover is faster during prometaphase and slower in metaphase, a process that is modulated by CDK1:CYCLIN A levels (Kabeche and Compton, 2013). Depletion of CYCLIN A results in premature stabilization of KT-MT interactions in prometaphase and increases the rate of chromosome segregation errors when compared to control cells (Kabeche and Compton, 2013). A comprehensive analysis of CDK1:CYCLIN A substrates was recently conducted and, as expected for a general mitotic regulator, numerous mitotic targets were identified (Dumitru et al., 2017). Given the typical CYCLIN A degradation profile as cells reach metaphase, it is proposed that CDK1:CYCLIN A contributes to the indiscriminate regulation of KT-MT end-on attachments by maintaining these interactions in a relatively unstable state. The essential role of MT turnover in the fidelity of chromosome segregation is further highlighted by the correlation found between lower MT turnover rates and increased mis-segregation events in human cancer cell lines (Bakhoum et al., 2009b). Accordingly, increasing MT turnover rates by overexpression of the MT-depolymerases KIF2B or MCAK can significantly suppress the frequency of chromosome mis-segregation events in a chromosomally unstable human cell line (Bakhoum et al., 2009a). Nevertheless, the stability of KT-MT interactions gradually increases as mitosis progresses. This increase in the steady-state number of attached MTs probably reflects the need to build robust K-fibers to drive chromosome segregation. Hence, KT-MT end-on attachment dynamics is maintained within a precise range during different stages of mitosis to ensure faithful genome transmission.

Remarkably, the establishment of appropriate bioriented attachments further stabilizes KT-MT interactions. In a classical set of experiments using microneedles to induce syntelic KTs in insect spermatocytes, Nicklas and Koch found that MTs attached to these KTs were unstable unless tension was applied with a microneedle pulling the chromosome mass towards the opposite spindle pole (Nicklas and Koch, 1969). Later, it was found that the establishment of tension was correlated with an increase in the number of MTs bound to KTs, thus suggesting that tension decreases the rate of MT detachment (King and Nicklas, 2000). Consistent with these initial observations, work *in vitro* using beads decorated with purified yeast KT components demonstrated that, when tension was applied to a reconstituted bead-MT end-on attachment, the lifetime of the attachment was increased (as in a “catch bond” interaction) (Akiyoshi et al., 2010). Moreover, tight binding of NDC80c-containing beads to a MT tip was shown to slow down MT depolymerization when beads were placed under an optical trap (Umbreit et al., 2012; Volkov et al., 2018). Therefore, tension-dependent models for the stabilization of KT-MT interactions envision that correctly bioriented attachments are able to build tension across the inter-chromatid axis as a result of pulling forces exerted towards opposite directions. This causes a reduction in the rate of detachment and stabilizes KT-MT end-on binding. However, the mutual dependence between stable attachment formation and tension raises the question of how initial bioriented attachments are formed (a conundrum known as the “initial problem of biorientation”) (Zhang et al., 2013; Kalantzaki et al., 2015; Tubman et al., 2017; Edelmaier et al., 2020). Initial low-tension amphitelic KT-MT attachments have to be converted into high-tension attachments, rather than relapse to unattached. A delay in the process leading to MT detachment could favor this transition (Tubman et al., 2017). Mathematical models propose that an increase in the lifetime of attachments can occur if MT release requires multiple phosphorylation events on key KT substrates, a plausible scenario *in vivo* (Tubman et al., 2017). Additionally, a delay in MT detachment could be provided by mechanisms ensuring stabilization of low tension monotelic attachments (only one sister KT is bound end-on to MTs), such as chromokinesin-mediated polar ejection forces (PEFs) (Cane et al., 2013; Drpic et al., 2015) or motor protein-dependent lateral interactions at the sister KT (Kuhn and Dumont, 2017; Edelmaier et al., 2020). Both activities generate forces that are balanced by a pulling force resulting from MT depolymerization at the attached KT-MT interface (Khodjakov and Rieder, 1996). This can potentially lead to rising tension levels at the bound KT which eventually increases the lifetime of attachments before biorientation (Akiyoshi et al., 2010). Together, these mechanisms create an opportunity for low tension amphitelic attachments to be gradually stabilized.

A tension-dependent modulation of KT-MT interactions also provides a targeted approach to deal with error correction in a chromosome-specific manner (Yoo et al., 2018). The underlying molecular players include well established KT-localized MAPs directly involved in supporting or modulating KT-MT end-on attachments, such as NDC80c, SKAc, KIF2B or MCAK. Moreover, these MAPs are under the regulation of AURORA B kinase, a canonical component of the error correction machinery (Krenn and Musacchio, 2015; Lampson and Grishchuk, 2017). Given the known functions of its KT substrates, AURORA B is expected to trigger either the release of MTs or promote MT depolymerization, hence assuming a pivotal role in the error-correction mechanism. Importantly, compelling evidences indicate that the function of AURORA B in error-correction is regulated in a tension-dependent manner, hinting for a role of AURORA B as a key intermediary player in a tension sensing mechanism (Krenn and Musacchio, 2015; Lampson and Grishchuk, 2017; Broad and DeLuca, 2020).

Additional regulatory mechanisms promote the efficient correction of erroneous interactions in an AURORA B-independent manner. These involve KT-localizing proteins such as chTOG and the RZZ complex (RZZc). chTOG, a conserved MT polymerase that requires NDC80 to localize to KTs, causes MT detachment specifically from KTs under low tension. On the other hand, chTOG is also able to stabilize tension bearing attachments. Due to these dual effects, chTOG is regarded as an intrinsic error correction mechanism (Miller et al., 2016; Herman et al., 2020). An unexpected role has also been attributed to the RZZc in inhibiting the MT-binding activity of NDC80 ^48–51^. Despite limited knowledge on the regulatory and mechanistic details behind both chTOG and RZZc functions in error correction, these molecular elements have been proposed to cooperate with the canonical AURORA B function in preventing stabilization of erroneous KT-MT end-on interactions.

Mitotic kinases other than AURORA B (and CDK1) also actively engage in the control of dynamic interactions between KTs and MTs and ultimately promote higher fidelity of chromosome segregation. MPS1, a critical orchestrator of SAC signaling, is also implicated in error-correction by phosphorylating key players involved in the KT-MT attachment process. Although not consistently observed in different human cell lines (Hewitt et al., 2010; Maciejowski et al., 2010; Santaguida et al., 2010), MPS1 has been proposed to accelerate the accumulation and activity of AURORA B at centromeres (Jelluma et al., 2008; van der Waal et al., 2012). Moreover, MPS1 can directly phosphorylates NDC80 (Sarangapani et al., 2021) and the SKAc (Maciejowski et al., 2017) to weaken KT-MT interactions independently of AURORA B. PLK1, another key Ser/Thr mitotic kinase, is also crucial for the regulation of KT-MT attachments. However, unlike AURORA B and MPS1, PLK1 activity has been shown to promote both destabilization and stabilization of KT-MT interactions (Godek et al., 2015). How these opposite outcomes are selectively orchestrated by PLK1 remains largely elusive.

In the subsequent sections, we focus on the regulatory functions of PLK1 and AURORA B towards KT-MT attachment stability and address how these functions are timely controlled during mitosis. Additionally, we discuss the recently proposed function for the RZZc in the inhibition of stable KT-MT end-on interactions. Altogether, this intricate network of centromere- and KT-localized regulators fine-tunes KT-MT end-on attachments to limit the formation of erroneous interactions and to stabilize amphitelic attachments, thereby ensuring the maintenance of genomic integrity.

### AURORA B-dependent Mechanisms

**FIGURE 2:**
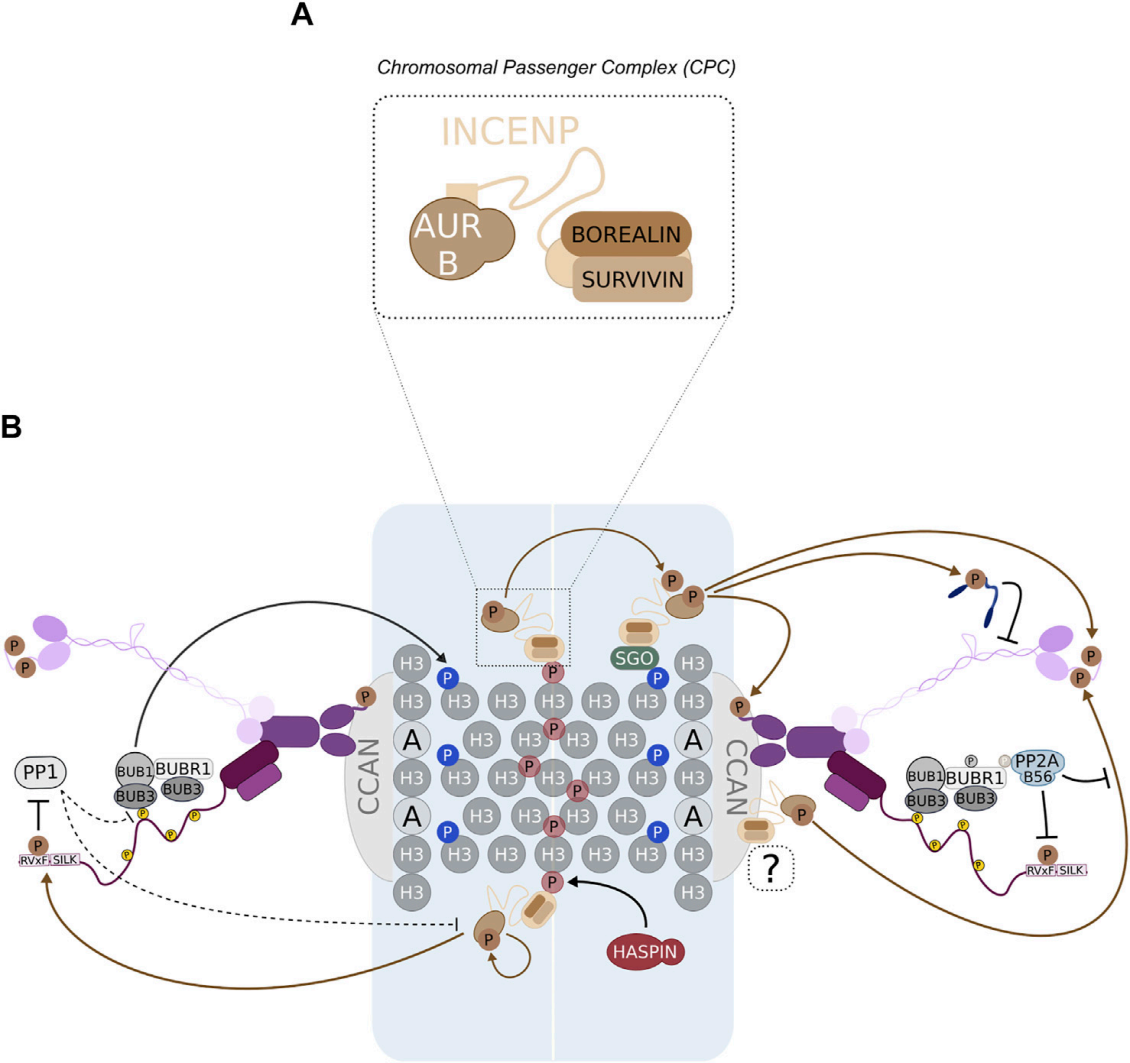
AURORA B localizes at multiple sites along the centromere-KT axis. **(A)** AURORA B kinase associates with INCENP, BOREALIN and SURVIVIN to assemble the chromosomal passenger complex (CPC). **(B)** The conspicuous centromeric localization of the CPC relies on the interaction of SURVIVIN and BOREALIN with either inner centromeric phospho-H3-Thr3 or KT-proximal SHUGOSHIN1/2 (SGO), respectively. H3-Thr3 is phosphorylated by HASPIN, while KNL1-associated BUB1 phosphorylates H2A-Thr120 to recruit SGO (note that nucleosomes are represented either as “H3” or “A” to distinguish between canonical H3- or CENP-A-containing nucleosomes). It has also been proposed that a pool of AURORA B is present at KTs, although the KT receptor for this pool remains unknown (“?”). Importantly, the prominent accumulation of CPC at centromeres supports prompt activation of AURORA B through *trans* auto-phosphorylation. Additionally, AURORA B phosphorylates INCENP to fully activate the CPC complex. Active centromeric and KT AURORA B phosphorylate critical substrates with key roles in KT-MT attachment stabilization, namely the NDC80c (N-terminal tail in NDC80), the SKAc and KNL1 (PP1-docking motif). The activity of AURORA B is likely counteracted by PP2A:B56 during early mitotic stages and by PP1 at later stages.

The regulation of KT-MT end-on attachment stability is intimately linked to the activity of AURORA B kinase. This kinase belongs to the Aurora family of proteins which additionally includes AURORA A in higher eukaryotes. In mammals, a third member, AURORA C is also present (Carmena et al., 2012a). AURORA B is an essential mitotic kinase that integrates the chromosomal passenger complex (CPC), a multi-subunit assembly that additionally comprises INCENP, BOREALIN and SURVIVIN (Figure 2A). The complex is assembled with the largest subunit INCENP bridging two modules, each one assigned with a different function. AURORA B binds to the IN-box domain at the C-terminal end of INCENP and provides the catalytic activity of the CPC. In turn, the N-terminal end of INCENP mediates binding to BOREALIN and SURVIVIN that together drive the subcellular localization of the complex (Krenn and Musacchio, 2015; Carmena et al., 2012a). The CPC concentrates prominently at centromeres until metaphase, although a KT-localized pool of AURORA B has also been observed in early mitotic cells (Figure 2B) (Krenn and Musacchio, 2015; Broad and DeLuca, 2020). In addition, the CPC is able to associate with MTs through INCENP and BOREALIN MT binding domains (Tseng et al., 2010; van der Horst et al., 2015; Funabiki, 2019; Trivedi et al., 2019). Following anaphase onset, the CPC is targeted to the spindle midzone where it controls cytokinesis-related events (Carmena et al., 2012a).

#### AURORA B Centromere/KT Localization

The accumulation of the CPC at the centromeric region involves two pathways that rely on histones H3 and H2A (Figure 2B). These recruitment arms are regulated by HASPIN and BUB1 kinases, which phosphorylate histone H3 on threonine 3 (H3-pThr3) and histone H2A on threonine 120 (H2A-pThr120), respectively. Once phosphorylated, H3-pThr3 binds directly to SURVIVIN and H2A-pThr120 recruits BOREALIN indirectly via SHUGOSHIN1/2 (SGO1/2) proteins, restricting the CPC to the centromeric region (Broad and DeLuca, 2020). The use of specific antibodies to visualize both phospho-histone marks made possible to pinpoint the subcellular position of the CPC within the KT-centromere axis. These studies show that H3-pThr3 mediates the recruitment of the CPC to the inner centromere, whereas H2A-pThr120 localizes the CPC outwards, at the KT-proximal centromere (Figure 2B) (Broad et al., 2020; Hadders, 2020; Liang et al., 2020). Considering that each phospho-histone mark was sufficient to recruit AURORA B to ectopic sites in human cells, these results indicate that each recruitment arm operates independently to localize distinct CPC populations. In agreement with this, experiments where histone phospho-modifications were individually suppressed demonstrated that either H3-pThr3 or H2A-pThr120 are sufficient to recruit the respective CPC population to centromeres (Broad et al., 2020; Hadders, 2020; Liang et al., 2020). Nevertheless, an interplay between both recruitment arms appears to be required to specifically enrich the CPC at the centromeric region and promote AURORA B auto-activation. It has been recently proposed that such well-confined concentration of CPC molecules may be important for the precise regulation of SAC signaling (Liang et al., 2020), although another study diverges on this matter (Hadders, 2020). On the other hand, in higher eukaryotes, the maintenance of normal levels of centromeric AURORA B is regarded as crucial to regulate KT-MT error correction and prevent chromosome mis-segregation (Broad et al., 2020; Hadders, 2020; Liang et al., 2020; Abad et al., 2021). Whether each CPC pool controls specific players involved in error correction, as suggested by the ability of individual phospho-histone marks to ensure faithful chromosome segregation, remains to be determined.

The use of antibodies that specifically recognize activating phospho-epitopes on AURORA B and INCENP consistently highlight the presence of an active pool of the CPC at mammalian KTs (Posch et al., 2010; DeLuca et al., 2011; Caldas et al., 2013; Broad et al., 2020). The subsequent spatiotemporal characterization of active CPC localization revealed that it is recruited to KTs and centromeres during early mitosis and gradually shifts to a predominantly centromeric accumulation in late prometaphase and metaphase (DeLuca et al., 2011; Broad et al., 2020). In budding yeast, the CPC is likely to be recruited to KTs through an interaction between SLI15/INCENP and both CTF19/CENP-P and MCM21/CENP-O (Fischböck-Halwachs et al., 2019; García-Rodríguez et al., 2019). Together with OKP1/CENP-Q and AME1/CENP-U, these proteins assemble the COMA complex at inner KTs and, therefore, can provide a platform for the localization of the CPC independently of centromeric cues. Both CTF19 and MCM21 contain RING finger, WD repeat and DEAD-like helicases (RWD) domains, a feature shared by other KT components to mediate protein-protein interactions between discrete protein complexes (Schmitzberger and Harrison, 2012; Petrovic et al., 2014; Schmitzberger et al., 2017). Accordingly, the C-terminal RWD domain in CTF19 was shown to be required for SLI15 interaction *in vitro* (Fischböck-Halwachs et al., 2019). A role for the COMA complex in ensuring CPC localization and function at KTs also anticipates an explanation for the lack of apparent mitotic defects in a strain carrying a mutated version of SLI15 unable to support the centromeric localization of CPC (Campbell and Desai, 2013). Altogether, these results suggest that, at least in budding yeast, the centromere localization of the CPC is largely dispensable for its functions in error correction and SAC signaling. Instead, these functions are probably executed by the CPC associated with the inner KT. As discussed in the previous paragraph, this does not appear to be the case for mammalian cells, where the centromeric recruitment of CPC is necessary for error-free mitosis (Hadders, 2020; Liang et al., 2020). It is important to mention that the KT-MT interface varies significantly between yeasts and human cells, namely in the number of MTs that comprise KT-fibers. As each KT in budding yeast binds only a single MT, no merotelic attachments arises. Nevertheless, whether a similar inner KT-dependent recruitment pathway for the CPC exists in vertebrates and/or if other proteins are involved in CPC loading to KTs awaits experimental confirmation.

#### The Role of AURORA B in KT-MT Attachment Destabilization

AURORA B has a fundamental function in safeguarding the accuracy of chromosome segregation by regulating the dynamic interaction between KTs and MTs. The repeated cycles of MT attachment and detachment constitute an essential feature for the correction of improper KT-MT end-on attachments that are formed in early mitosis and AURORA B is key in the error correction process. The gene encoding the kinase, *ipl1*, was identified in a genetic screen for genes required for maintenance of ploidy in *S. cerevisiae* (Chan and Botstein, 1993). Following its discovery, different studies dissected the roles of IPL1/AURORA B during mitosis. Initial experiments in *S. cerevisiae* unveiled a crucial function of IPL1 in promoting accurate KT-MT attachments (Biggins et al., 1999; Tanaka et al., 2002). Similar observations were made in mammalian cells, where selective repression of AURORA B activity after treatment with RNAi or small-molecule inhibitors led to chromosome alignment defects and an accumulation of erroneous syntelic and merotelic KT-MT attachments (Ditchfield et al., 2003; Hauf et al., 2003; Cimini et al., 2006).

**FIGURE 3:**
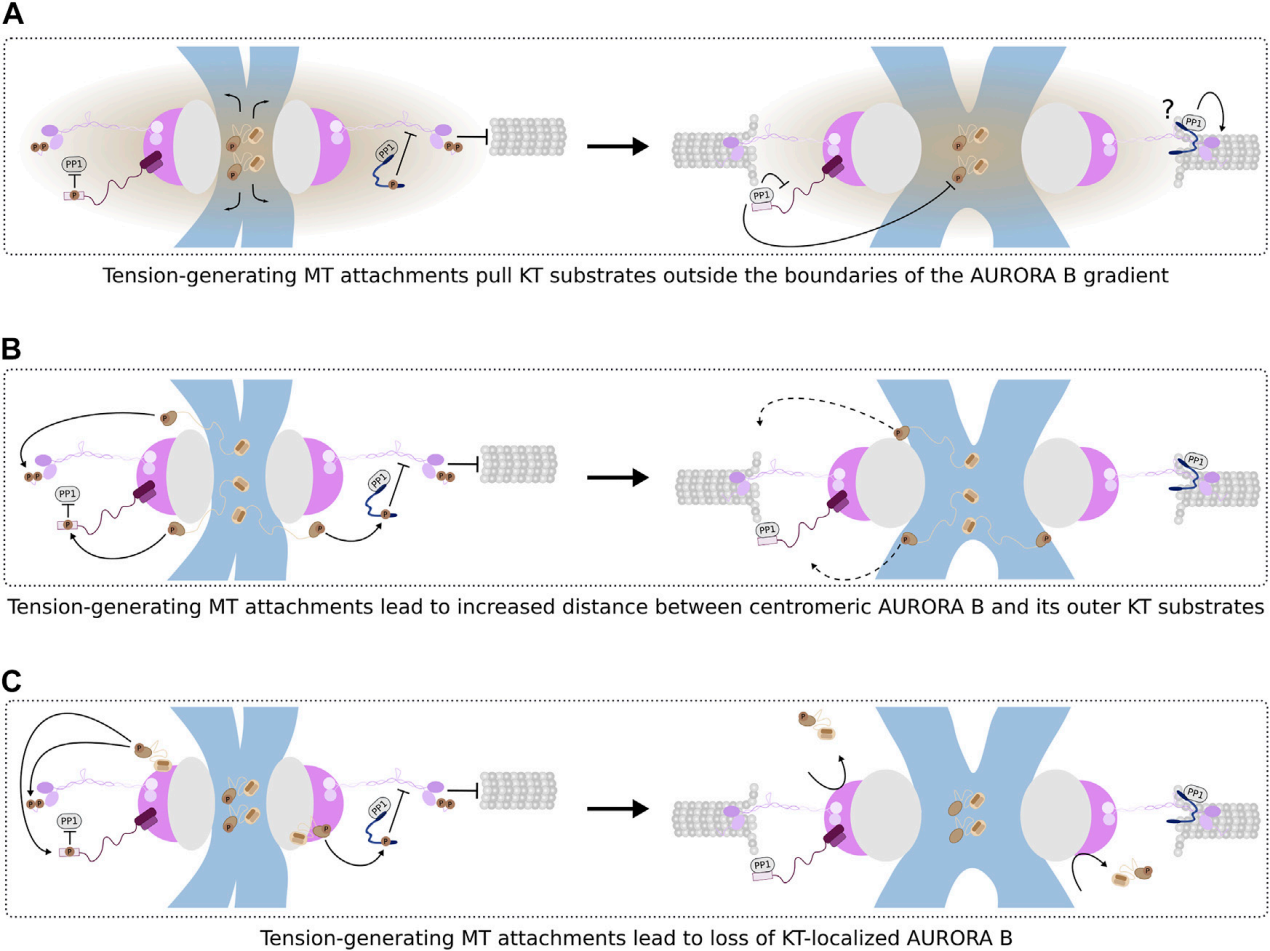
AURORA B-dependent regulation of KT-MT attachment stability. **(A), B)** Spatial positioning models for AURORA B function based on a **(A)** diffusible phosphorylation gradient or **(B)** physical distance between the kinase and substrates. Both models predict that the increase in inter-KT tension as a result of correct amphitelic KT-MT attachments pulls KT substrates away from the zone of influence of active AURORA B. **(C)** Model for the regulation of KT-localized AURORA B function. In this model, the gradual increase in KT-MT attachment stability evicts AURORA B from KTs which, in turn, further stabilizes KT-MT interactions. Dephosphorylation of AURORA B substrates like NDC80, KNL1 and the SKAc contributes to stabilize load-bearing end-on attachments. Interestingly, the SKAc recruits PP1 to KTs, but it remains elusive whether such PP1 pool contributes to the stabilization of KT-MT interactions (“?”).

Time-lapse visualization of chromosome-MT interactions in AURORA B-inhibited human cells confirmed that improper attachments result from the premature stabilization of KT-MT interactions. Importantly, syntelic KT-MT attachments could be corrected upon re-activation of AURORA B following inhibitor washout, indicating that AURORA B kinase has a key role in promoting error correction (Lampson et al., 2004). Interestingly, the correction of syntelic interactions appeared to occur by disassembling KT-fibers (Lampson et al., 2004) and not by releasing MTs from KTs, as initially proposed for the error correction process in the budding yeast (Tanaka et al., 2002). Instead, MT detachment is probably required to resolve merotelic attachments (Cimini et al., 2006). In contrast to syntelic attachments which are corrected early in mitosis, merotelic interactions can still be found at aligned chromosomes (Gregan et al., 2011). Correction of merotelic attachments requires that KTs release MTs extending from the distal pole while the connections to correct MTs are maintained. Recent data suggests that the distinct levels of inter-KT tension associated with each attachment configuration regulate how AURORA B impacts the turnover of KT-MT interactions (Figures 3A–C). Using optogenetics to recruit and activate AURORA B with high spatiotemporal control, it was show that at syntelic KT pairs, which experience low tension, MT detachment does not occur immediately upon kinase activation and the chromosome is pulled closer to the spindle pole in a MT-depolymerization dependent manner (Chen et al., 2021). Importantly, MT depolymerization still allows AURORA B activity to prevent accumulation of erroneous interactions by bringing chromosomes towards the spindle pole where the combined actions of KT/centromere-localized AURORA B and centrosome-localized AURORA A promote MT detachment from KTs (Chmátal et al., 2015; Ye et al., 2015; Lampson and Grishchuk, 2017). Under increased tension, optogenetic-driven activation of AURORA B results in MT release from KTs. It will be interesting to determine whether this mechanism also underlies the correction of merotelic KTs (Chen et al., 2021) without the ectopic recruitment of AURORA B (as the kinase is expected to be silenced in high tension attachments). If so, it becomes imperative to understand how AURORA B substrates at KTs/centromeres are differently affected by distinct levels of inter-KT tension so that MT depolymerization or MT release are specifically triggered.

#### AURORA B Phosphorylates the NDC80c

A large number of KT- and centromere-localized AURORA B substrates have been reported (Krenn and Musacchio, 2015; Carmena et al., 2012a; Kettenbach et al., 2011; Lampson and Cheeseman, 2011). Many of these substrates contain a consensus sequence, Arg/Lys-*X*-Ser/Thr-*h* (*X* is any amino acid and *h* is an hydrophobic or aromatic amino acid), that is recognized by AURORA B (Kettenbach et al., 2011; Cheeseman et al., 2002). Considering its primary function in the establishment of KT-MT end-on attachments, the NDC80c represents a key AURORA B target in the error correction mechanism (Figure 2B). It was demonstrated that AURORA B phosphorylates the unstructured N-terminal tail of NDC80 on multiple residues *in vitro*, a feature conserved across evolution (Cheeseman et al., 2006; DeLuca et al., 2006; Ciferri et al., 2008; Akiyoshi et al., 2009). In mammalian cells, a subset of these *in vitro*-mapped AURORA B phosphosites were shown to be present at KTs preferentially during early mitosis, then decreasing as KTs efficiently aligned at the metaphase plate (DeLuca et al., 2011). Phosphorylation of the N-terminal tail likely abrogates the electrostatic interactions between this region and the negatively charged C-terminal ends of TUBULIN monomers, thereby reducing the MT-binding activity of the NDC80c (Ciferri et al., 2008; Miller et al., 2008). AURORA B promotes the dissociation of the NDC80c from MTs *in vitro* (Cheeseman et al., 2006; Ciferri et al., 2008) and NDC80 tail mutants with increasing numbers of phospho-mimetic substitutions are unable to support stable MT end-on attachments in mammalian cells (Guimaraes et al., 2008; Alushin et al., 2012; Zaytsev et al., 2014; Yoo et al., 2018). Consequently, the number of MTs attached to KTs decreases significantly, arguing that AURORA B-dependent phosphorylation of NDC80 underlies error correction. Expectedly, expression of an phospho-null NDC80 tail mutant in mammalian cells led to an accumulation of improper KT-MT attachments that compromised chromosome segregation (DeLuca et al., 2011; Sundin et al., 2011).

Compelling evidence suggests that the MT-binding activity of the NDC80 tail contributes to the overall MT-binding affinity of the NDC80c. Additionally, the N-terminal tail could provide the KT with load bearing activity through a non-mutually exclusive function in mediating the clustering of adjacent NDC80c into oligomeric structures. The incubation of low concentrations of fluorescently-tagged NDC80 complexes with MTs revealed a noticeable organization of NDC80c molecules in clusters, suggesting that binding to the MT surface may occur in a cooperative manner (Ciferri et al., 2008). Subsequent cryo-EM studies with purified components demonstrated that the removal of the NDC80 tail prevented the formation of larger clusters on the surface of MTs, which supports the notion that the N-terminal tail mediates NDC80c oligomerization (Alushin et al., 2010; Alushin et al., 2012). Interestingly, introduction of phospho-mimetic substitutions on the N-terminal tail led to a decrease in the number of NDC80 complexes *per* cluster. This suggests that AURORA B is able to regulate the stability of KT-MT end-on attachments by modulating the NDC80 tail-dependent cooperative binding of NDC80c to MTs (Alushin et al., 2010; Alushin et al., 2012). A detailed analysis further assigned different functional roles to two segments within the N-terminal tail, both of them regulated by AURORA B. While a truncated NDC80 tail fragment encompassing the C-terminal region proximal to the globular head displayed MT-binding activity similar to the full length tail, a fragment comprising only the N-terminal half of the NDC80 tail failed to bind to MTs. Interestingly, mimicking phosphorylation of AURORA B residues in the latter fragment impaired the formation of NDC80c clusters (Alushin et al., 2012). Collectively, these results suggest that the N-terminal tail of NDC80 contains two regions whose regulation by AURORA B controls cluster stability or MT attachment affinity. However, this model is challenged by observations supporting that, regardless of the position of each residue, the sequential phosphorylation of the unstructured tail gradually decreases the MT-binding affinity of the NDC80c (Zaytsev et al., 2015). The same study reported that the interaction between the unstructured tail and an adjacent NDC80c is predicted to be of weak nature and insensitive to AURORA B-dependent phosphorylation of the NDC80 tail, arguing against a model where NDC80 complexes bind MTs as oligomeric structures (Zaytsev et al., 2015). The conflicting results demand further experimental evaluation of the biochemical properties of the NDC80c and additional *in vivo* characterization of how AURORA B impacts on NDC80c activity towards KT-MT end-on attachments.

The specific context of the KT-MT interface, where interactions with dynamic MTs must be well coordinated, has to be taken into account when describing NDC80c regulation. In that respect, it is important to consider that KTs experience pulling/pushing forces when bound to the plus end of dynamic MTs. As such, the assembly of small NDC80c clusters might prove to be critical for KTs to be able to maintain interactions with dynamic MTs (Volkov et al., 2018). An AURORA B-dependent phosphorylation of the NDC80 tail could be required to regulate the binding activity of NDC80c clusters, ultimately modulating the ability of the KT to couple with dynamic MTs. Recently, it was shown that bead-bound trimerized NDC80 complexes lacking the N-terminal tail or bearing phospho-mimetic mutations on AURORA B target residues were competent to bind to the MT lattice in the absence of any pulling or resisting force (Huis In 't Veld et al., 2019). The ability to bind to the MT surface is probably dictated by the intact CH domain of NDC80. However, both mutant complexes could not track with depolymerizing MT ends when placed under force to mimic load and consequently detached from the MT tip (Huis In 't Veld et al., 2019). These results suggest that the N-terminal tail in NDC80 is required for force-coupling to dynamic MTs and that AURORA B modulates this interaction. Such a model is in agreement with earlier observations that support a role for the NDC80 tail in maintaining points of contact with curved protofilaments in depolymerizing MTs (Alushin et al., 2010). Intriguingly however, the data collected *in vitro* is not in line with recently reported *in vivo* data proposing that AURORA B-dependent phosphorylation of NDC80 affects the KT’s ability to move along polymerizing (anti-poleward movement) but not depolymerizing (poleward movement) MTs (Long et al., 2017). These discrepancies highlight the complex nature of KTs as it is possible that, *in vivo*, additional KT components compensate for a decrease in NDC80c binding to depolymerizing MTs and, hence, prevent full KT detachment. Additionally, the KT composition at the MT binding interface may differ between both scenarios.

#### AURORA B Regulates Numerous Effectors of KT-MT Destabilization

In addition to the NDC80c, the remaining KMN components were also reported as AURORA B substrates (Welburn et al., 2010). Despite being required for full MT-binding activity of a reconstituted KMN complex *in vitro*, both MIS12c and KNL1 do not appear to be directly involved in supporting end-on contacts between KTs and MTs (Cheeseman et al., 2006; Caldas et al., 2013; Espeut et al., 2012; Audett et al., 2022; Bajaj et al., 2018). Thus, AURORA B-mediated regulation of each complex controls distinct aspects of KT function. During mitosis, AURORA B phosphorylates DSN1 to enhance the interaction between MIS12c and the inner KT component CENP-C (Figure 2B) (Petrovic et al., 2016; Dimitrova et al., 2016). AURORA B also phosphorylates KNL1 in its N-terminal RVSF motif to inhibit the recruitment of PP1 phosphatase to the outer KT (Figure 2B) (Welburn et al., 2010; Liu et al., 2010). As mitosis progresses and KT-MT end-on attachments are gradually stabilized, phosphorylation of the RVSF motif in KNL1 is alleviated which results in increased levels of PP1 at KTs (Figures 3A–C) (Nijenhuis et al., 2014). Once recruited to the outer KT, PP1 is expected to dephosphorylate AURORA B substrates to strengthen KT-MT interactions (Liu et al., 2010; Saurin, 2018; Moura and Conde, 2019).

AURORA B targets different MAPs, including NDC80c, DAM1c or SKAc, that are required to physically link KTs to MTs (Figure 2B). DAM1c or SKAc associate with the NDC80c and this interaction is weakened by AURORA B-dependent phosphorylation of the former complexes, leading to the destabilization of KT-MT end-on attachments (Lampert et al., 2010; Tien et al., 2010; Chan et al., 2012). Additionally, AURORA B regulates other MAPs that can modulate the dynamic behavior of the MT population within KT-fibers. For instance, AURORA B phosphorylates KIF2B and MCAK, 2 MT depolymerases with key roles in the regulation of MT turnover at KTs (Godek et al., 2015). The activities of both depolymerases are temporally segregated, with KIF2B and MCAK exerting their functions during prometaphase and metaphase, respectively (Bakhoum et al., 2009a). The sequential activation of MT depolymerases correlates with the profile of AURORA B activity. High AURORA B activity at prometaphase is required for KIF2B recruitment to and function at KTs (Bakhoum et al., 2009a). Interestingly, MCAK regulation is more complex as it has been shown that the recruitment, but not the activity of MCAK requires AURORA B (Ritter et al., 2016). During prometaphase, MCAK co-localizes with AURORA B at the centromeric region, whereas in metaphase MCAK localizes to the KT-proximal region (Andrews et al., 2004). This localization pattern has been proposed to rely on the interaction of MCAK with SGO2, which follows a similar distribution along the centromere-KT axis (Ritter et al., 2016). Moreover, the recruitment of MCAK to centromeres depends on AURORA B activity and several phosphorylation sites have been mapped in MCAK N-terminus *in vitro* (Andrews et al., 2004; Lan et al., 2004; Huang et al., 2007; Tanno et al., 2010; McHugh et al., 2018). Despite its influence on MCAK localization, AURORA B-dependent phosphorylation decreases the affinity of MCAK for the MT lattice *in vitro*, suggesting that initial recruitment of MCAK to centromeres is not accompanied by a concomitant increase in MCAK-dependent MT depolymerization (McHugh et al., 2018). The release from AURORA B-dependent inhibition probably requires MCAK to shift its position further away from centromeric AURORA B (Andrews et al., 2004). This fine control of MCAK activity towards KT-MT attachments is consistent with a more prevalent role for MCAK in the regulation of MT turnover during metaphase, in particular to resolve improper merotelic attachments that might still remain uncorrected (Bakhoum et al., 2009a). While it is expected that modulation of NDC80 MT binding affinity has a larger impact on the stability of KT-MT end-on attachments compared to modulation of KIF2B and MCAK, mis-regulation of the activity of MT depolymerases can have profound consequences on mitotic fidelity (Bakhoum et al., 2009a; Bakhoum et al., 2009b).

#### AURORA B Activity vs. KT Tension

Despite the growing understanding of the components that participate in the error correction mechanism governed by AURORA B, it is still under debate how the distinct AURORA B pools are regulated to coordinate their functions towards end-on attachment formation. While AURORA B does not affect initial KT-MT lateral interactions (Kalantzaki et al., 2015; Shrestha et al., 2017), which are mediated by KT-localized MAPs like DYNEIN, CENP-E or CENP-F (Itoh et al., 2018; Sang and Walczak, 2010), AURORA B-dependent phosphorylation of proteins directly involved in KT-MT end-on interactions has to be suppressed at some point to allow KTs to maintain connections to dynamic MTs (Figures 3A–C). Interestingly, all AURORA B substrates identified so far seem to lack a clear motif that mediates the interaction with the kinase. This contrasts with another kinase, POLO/PLK1, that harbors a substrate-targeting domain known as Polo-box domain (PBD) (discussed below). Therefore, AURORA B function is probably limited by the physical distance to its substrates. This distance could reflect the degree of opposite pulling forces exerted by MTs on sister KTs, which is expected to be higher for correctly bioriented KT-MT attachments (Figures 3A,B). Such correlation may be crucial to regulate how the major centromeric AURORA B pool discriminates between proper and mal-oriented KT-MT interactions and explain why tension stabilizes attachments (Akiyoshi et al., 2010; Tanaka et al., 2002). This model was substantiated by experiments showing that a FRET sensor sensitive to AURORA B activity was differently phosphorylated depending on whether it was artificially targeted to the centromere- or outer KT-proximal regions (Liu et al., 2009). At properly bioriented KTs, a more distantly localized MIS12-targeted sensor was significantly less phosphorylated than a CENP B-targeted one, arguing that stabilization of KT-MT end-on attachments results from the spatial separation of centromeric AURORA B from its outer KT substrates (Lampson and Grishchuk, 2017; Lampson and Cheeseman, 2011). According to this model, the accumulation of CPC complexes at centromeres promotes the auto-activation of AURORA B and an increase in the levels of AURORA B-dependent phosphorylation at this location. Then, a diffusible phosphorylation gradient is established across the centromere-KT axis as a result of decreasing concentration of CPC molecules from the centromere outwards (Figure 3A). As bioriented KTs are formed, tension resulting from MT-based pulling forces brings the outer KT substrates out of the reach of the AURORA B gradient, favoring the stabilization of KT-MT end-on attachments (Figure 3A) (Lampson and Cheeseman, 2011; Lampson and Grishchuk, 2017). Although the spatial separation model is appealing, the existence of a phosphorylation gradient remains a controversial hypothesis (Krenn and Musacchio, 2015; Broad and DeLuca, 2020).

A similar FRET-based approach revealed that AURORA B substrates within the KMN network (KNL1, DSN1 and NDC80) are differently phosphorylated as KTs attach MTs, indicating that AURORA B activity discriminates between substrates separated by small distances (assuming that DSN1 co-localizes with NDC80c subunits SPC24:SPC25, the distance to NDC80 subunit is ∼45–60 nm) (Welburn et al., 2010). The ability to differentiate between closely located substrates would imply the existence of a steep phosphorylation gradient. Alternatively, the CPC anchored at the centromere may be able to deliver AURORA B activity to outer KT substrates without the need of a diffusible gradient (Figure 3B). It has been suggested that the central domain in INCENP can assume an extended conformation which could facilitate AURORA B-mediated phosphorylation in the absence of significant pulling forces. However, with increasing tension, INCENP extension is probably insufficient to deliver AURORA B to the outermost region where substrates like NDC80 and DAM1 or SKA complexes are located (Figure 3B) (Krenn and Musacchio, 2015). Consistent with this notion, a mutated version of INCENP bearing a shorter central domain precludes phosphorylation of outer KT but not of centromeric AURORA B substrates in chicken DT40 cells (Samejima et al., 2015). Moreover, high resolution microscopy demonstrated that the co-localization between AURORA B and NDC80c decreases with increasing inter-KT distances (Yoo et al., 2018). Therefore, this “dog leash” model, similarly to the spatial separation model, describes that the distance between the kinase and its substrates dictates whether phosphorylation occurs or not (Figures 3A,B). The dog leash model, however, does not anticipate the presence of a diffusible phosphorylation gradient to explain how substrates are differently phosphorylated by AURORA B.

Building on the observation that a pool of active AURORA B exists at KTs until late prometaphase, a third model has been recently proposed (Broad and DeLuca, 2020). This model posits that AURORA B is directly recruited to KTs where it phosphorylates crucial outer KT components. Once correct KT-MT attachments are formed and tension is established, the AURORA B pool at KTs is lost (Figure 3C). The same model envisages that KNL1 may constitute a central part in the signaling mechanism that regulates loss of AURORA B (Broad and DeLuca, 2020). It has been recently shown that KNL1 undergoes structural rearrangements in response to the formation of tension-exerting KT-MT attachments (Roscioli et al., 2020). In particular, the N-terminal region of KNL1 assumes an extended configuration at tensionless KTs and re-organizes into a packed arrangement once tension is established. Together with data indicating that KNL1 is required for AURORA B activity at KTs, presumably through KNL1-bound BUB1 (Caldas et al., 2013; Broad et al., 2020), these results suggest a possible role for KNL1 in providing a tension-sensing platform for AURORA B localization at KTs (Broad and DeLuca, 2020). Nonetheless, it is still unknown whether KNL1 directly recruits the CPC to KTs or if other mechanisms are present to control AURORA B binding to and eviction from KTs. Given the presence of multiple pools of AURORA B, it also remains elusive whether the KT- and centromere-localized AURORA B populations exhibit any crosstalk and if these share substrate phosphorylation.

Finally, multiple studies support a model for AURORA B regulation that postulates that kinase activity is stimulated upon binding of the CPC to MTs (Funabiki, 2019). According to this model, in low tension attachments, binding of CPC to tubulin could facilitate AURORA B-dependent phosphorylation of KT substrates that become less accessible upon engagement with the plus ends of MTs (ex, NDC80) (Funabiki, 2019). As tension develops with the establishment of correct attachments, AURORA B targets at KTs could become increasingly more inaccessible and, hence, less prone to being phosphorylated. Alternatively, AURORA B could be displaced from near its KT substrates via a “treadmill” translocation on polymerizing MTs bound to the trailing KT (Funabiki, 2019). However, a model for MT-bound AURORA B in KT-MT attachment regulation is disputed by evidence showing that CPC binding to MTs is suppressed by CDK1 (Funabiki, 2019) and that phosphorylation of several AURORA B substrates still occurs in mitotic cells deprived of MTs (ex, treated with nocodazole) (Broad and DeLuca, 2020).

A common assumption is that as correct attachment configurations are achieved, AURORA B activity at the outer KT is selectively downregulated. The readout for properly bioriented KT-MT attachments has for long been attributed to the inter-chromatid tension resulting from opposite MT-based pulling forces (Lampson and Grishchuk, 2017). According to the prevailing models discussed above, AURORA B activity towards KT-localized substrates declines as increasing tension removes them from the kinase zone of influence (Figures 3A–C). Importantly, it has been demonstrated that tension can be induced within KTs—intra-KT—or across sister KTs—inter-KT (Krenn and Musacchio, 2015). Intra-KT tension reflects a conformational change within the KT characterized by an increase in the separation between inner KT and outer KT components (Maresca and Salmon, 2010). This occurs when MTs are embedded within the KT following end-on attachment and has been proposed to contribute to SAC silencing (Joglekar, 2016). However, intra-KT tension presumably occurs at a single KT level and appears to be insensitive to inter-KT tension (Suzuki et al., 2014). Thus, intra-KT tension does not seem to integrate information from the attachment status of sister KTs which is required to discriminate between proper and incorrect KT-MT attachments. In contrast, inter-KT tension is responsive to the attachment status of sister KTs and could more efficiently regulate how AURORA B performs its role in error correction.

#### PP1 and PP2A:B56 Antagonize AURORA B Activity

In addition to tension-sensing mechanisms, substrate dephosphorylation is expected to balance AURORA B-mediated inhibitory activity at the KT-MT interface (Figure 2B) (Gelens et al., 2018; Moura and Conde, 2019). For instance, given the negative impact that phosphorylation of NDC80 N-terminal tail has on KT-MT attachment stability, efficient dephosphorylation is expected to follow to allow the establishment of stable interactions (DeLuca et al., 2011). During mitosis, most phosphorylation events are reversed by the combined activities of Ser/Thr phosphatases PP1 and PP2A (Saurin, 2018; Moura and Conde, 2019). Several studies have suggested that both PP1 and PP2A:B56—the main PP2A holoenzyme involved in KT-MT attachment regulation (Foley et al., 2011)—antagonize AURORA B and cooperate to ensure the stabilization of KT-MT end-on attachments (Liu et al., 2010; Posch et al., 2010; Foley et al., 2011; Suijkerbuijk et al., 2012; Xu et al., 2013a; Kruse et al., 2013; Shrestha et al., 2017).

PP2A:B56 localizes to KTs during prometaphase, when it presumably promotes stabilization of end-on attachments (Foley et al., 2011). Recruitment to KTs depends largely on association with BUBR1 (discussed in detail later) and precluding this interaction leads to severe defects in chromosome alignment due to unopposed AURORA B activity (Foley et al., 2011; Suijkerbuijk et al., 2012; Xu et al., 2013a; Kruse et al., 2013). Accordingly, depletion of PP2A:B56 results in increased phosphorylation of known AURORA B substrates (Foley et al., 2011; Suijkerbuijk et al., 2012). This argues in favor of a role for PP2A:B56 in limiting the level of AURORA B activity and in preventing systematic destabilization of KT-MT attachments (Foley et al., 2011; Suijkerbuijk et al., 2012; Xu et al., 2013a; Kruse et al., 2013). Whether PP2A:B56 directly regulates AURORA B activation status, as proposed for a centromeric pool of PP2A:B56 bound to SGO1/2 (Meppelink et al., 2015), or directly dephosphorylates critical AURORA B substrates at KTs like NDC80, remains to be thoroughly analyzed.

Remarkably, PP2A:B56 positively impacts on the KT recruitment of the other main mitotic phosphatase PP1 ^109^. PP1 is recruited to the outer KT via an interaction with specialized SILK/RVxF motifs present in the N-terminus of KNL1 (Figure 1B) (Audett et al., 2022; Bajaj et al., 2018; Liu et al., 2010). While AURORA B phosphorylates these motifs, thereby repressing PP1 recruitment to KTs, PP2A:B56 promotes PP1 localization by removing those inhibitory phosphorylations (Figure 2B) (Liu et al., 2010; Nijenhuis et al., 2014). Once at the outer KT, PP1 can balance AURORA B activity. Consistent with this hypothesis, mutation of PP1-binding motifs in KNL1 led to an increase in AURORA B-dependent phosphorylation of a KT-targeted sensor in KT pairs under tension (Liu et al., 2010). A similar AURORA B hyperactivation phenotype was observed when SDS22, a different KT adaptor for PP1, was depleted (Posch et al., 2010). Moreover, forcing PP1 binding to KNL1 in the absence of PP2A:B56 was sufficient to restrict AURORA B activity (Nijenhuis et al., 2014). Together, these results attribute a relevant role to PP1 in counteracting AURORA B activity toward KT substrates and in further promoting the stabilization of KT-MT end-on attachments (Saurin, 2018; Liu et al., 2010). This view was recently challenged by a study that supports a predominant function for PP2A:B56 and not PP1 in facilitating initial establishment of end-on attachments (Shrestha et al., 2017). Nevertheless, PP1, either bound to KNL1 or other KT scaffolds, including CENP-E, KIF18A, SKAc and ASTRIN, can still be important to support the maintenance of attachment stability at amphitelic attachments (Figure 3A) (Saurin, 2018; Moura and Conde, 2019).

### POLO/PLK1-Dependent Mechanisms

AURORA B-mediated destabilization of KT-MT end-on attachments has been regarded as the canonical pathway in error correction. However, additional mitotic kinases, including MPS1, CDK1 (see both above) and POLO/PLK1 also function to promote KT-MT attachment turnover (Godek et al., 2015). Similar to AURORA B, which modulates KT-MT end-on attachment stability in a chromosome-autonomous manner, PLK1 has been implicated in the local control of KT-MT interactions. PLK1 belongs to the Polo-like kinase sub-family of Ser/Thr protein kinases and phosphorylates target proteins on the consensus sequence motif Asn/Asp/Glu-*X*-Ser/Thr-*h* (where *X* is any amino acid and *h* is an hydrophobic amino acid except Pro) (Nakajima et al., 2003; Santamaria et al., 2011; Zitouni et al., 2014). The kinase has emerged as a crucial mitotic regulator, being required for diverse functions such as timely mitotic entry, centrosome maturation, mitotic spindle assembly, cohesin dynamics, KT-MT attachments, and cytokinesis (Sunkel and Glover, 1988; Llamazares et al., 1991; Schmucker and Sumara, 2014; Colicino and Hehnly, 2018).

#### Structural Features and Activation of PLK1

**FIGURE 4:**
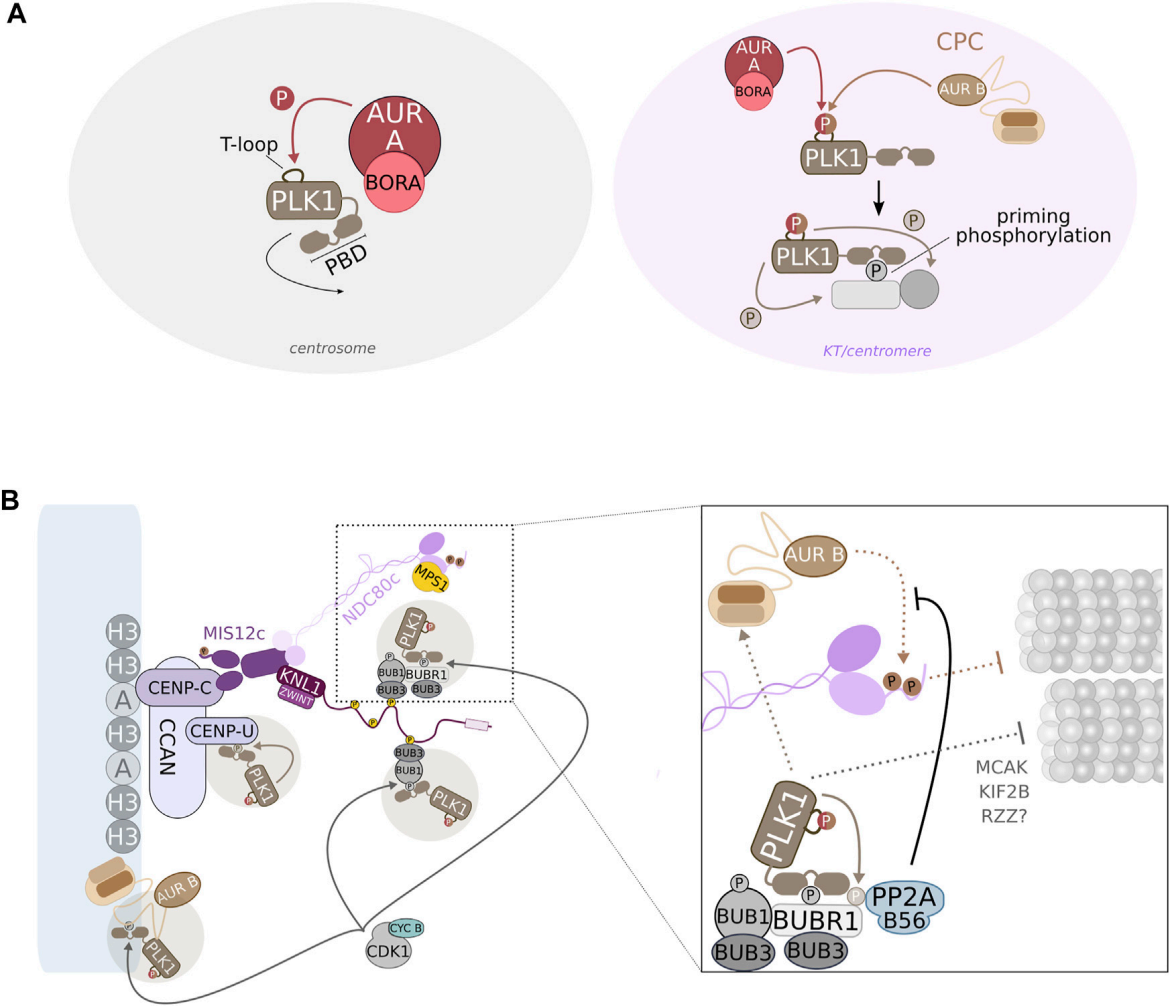
POLO/PLK1 controls the turnover of KT-MT attachments. **(A)** At late G_2_, AURORA A:BORA phosphorylates PLK1 on its N-terminal T-loop to activate the kinase. At this stage, activation of PLK1 by AURORA A:BORA occurs at centrosomes, but subsequent maintenance of PLK1 activity at centromeres/KTs is likely maintained by AURORA B (although a residual fraction of AURORA A:BORA at KTs may also promoting PLK1 activation). Phosphorylation of the T-loop in PLK1 probably relieves an intramolecular interaction that mutually inhibits the N-terminal kinase domain and the C-terminal PBD. This can potentially contribute to fully activate PLK1 by exposing the kinase domain and allowing the PBD to interact with pre-phosphorylated (priming phosphorylation) substrates/binding partners. **(B)** PLK1 is recruited to multiple locations along the centromere/KT axis. CDK1:CYC B-mediated priming phosphorylation of PLK1 binding partners provides a positional cue for the localization of PLK1. However, PLK1 can promote its own recruitment by self-priming (ex, CENP-U/PBIP1). At KTs, PLK1 regulates the turnover of KT-MT interactions by promoting both the stabilization—PLK1-dependent phosphorylation of BUBR1 triggers the recruitment of PP2A:B56 phosphatase that, in turn, counteracts AURORA B activity—and destabilization of attachments—PLK1-dependent phosphorylation of KIF2B and MCAK likely promote MT depolymerization. PLK1 may also promote the destabilization of KT-MT attachments through an RZZ-dependent mechanism.

The catalytic activity of PLK1 resides on its N-terminal kinase domain, whereas the intracellular localization of PLK1 relies on a C-terminal region comprising two adjacent ∼80-residue long segments, referred to as Polo-boxes, which fold together as a Polo-box domain (PBD) (Figure 4A) (Elia et al., 2003a). This region is highly conserved among the different Polo-kinase family members and functions as a single protein-binding unit to mediate the recruitment of PLK1 to specific sites during the cell cycle. Some PBD targets are themselves PLK1 substrates that, in this way, directly recruit a regulatory kinase to a position that favors their phosphorylation (Figure 4A). Importantly, the PBD typically recognizes a phosphorylated sequence motif on the POLO/PLK1 target that binds at a positively charged interface that is formed between the two Polo-boxes (Elia et al., 2003b; Cheng et al., 2003). Given that the PBD was isolated as a pThr-Pro-interacting domain, this indicated that CDK1 has a major role in priming target proteins for PLK1 interaction (Elia et al., 2003a). A comprehensive search for the optimal binding motif further identified the sequence Ser-pSer/pThr-Pro/*X* (where pSer/pThr denotes phosphorylated Ser or Thr and *X* is any amino acid) as the core consensus motif recognized by the PBD (Elia et al., 2003a). However, it has also been suggested that unconventional interactions exist between the PBD and target proteins. The majority of PLK1 targets during early-to-mid mitosis are expected to undergo priming phosphorylation in a CDK1:CYCLIN A or CDK1:CYCLIN B-dependent manner, but other priming kinases can also execute this task, thereby contributing to expand the repertoire of PBD-ligand interaction modes. Notably, in the presence of a PLK1 small-molecule inhibitor, the levels of PLK1 at KTs and centrosomes are severely reduced, suggesting that the kinase activity is required for its proper recruitment to these mitotic structures (Lénárt et al., 2007). This is in agreement with data supporting a role for PLK1 in generating its own docking site on PBIP1/CENP-U for subsequent PBD-dependent interactions (Figure 4B) (Kang et al., 2006). Notably, the PBD is also able to bind to target proteins in a phospho-independent manner, as in the case of BORA (Seki et al., 2008) and the POLO/PLK1 binding partner MAP205 (Archambault et al., 2008).

The N-terminal kinase domain of PLK1 affects the ability of the PBD to interact with its ligands, most likely due to intra-molecular interactions between both domains (Figure 4A) (Elia et al., 2003a; Elia et al., 2003b). Moreover, such interactions reciprocally obstruct both kinase and PBD functions (Mundt et al., 1997; Jang et al., 2002; Xu et al., 2013b). In fact, binding of PBD to a target protein can potentially relieve the PBD-kinase domain mutual inhibition that otherwise restricts the kinase activity and prevents full PLK1 function (Xu et al., 2013b). In addition, phosphorylation of the N-terminal kinase domain of PLK1 can also stimulate the release of the auto-inhibitory mechanism, thereby resulting in PLK1 activation (Figure 4A). This regulatory mechanism, whereby the kinase domain, namely its T-loop, is subjected to phospho-regulation, is common to several protein kinases. It has been shown that, during the G_2_-M transition, the phosphorylation of PLK1 on its activating T-loop coincides with a significant increase in kinase activity (Bruinsma et al., 2012). In particular, the phosphorylation of Thr210 in human PLK1 (or Thr201 in *Xenopus* PLX1) was shown to increase PLK1 catalytic activity by several-fold (Jang et al., 2002; Lee and Erikson, 1997; Qian et al., 1999). Subsequent studies demonstrated that phosphorylation of Thr210/Thr201 occurs *in vivo* and is required for PLX1/PLK1 activation before mitotic entry (Seki et al., 2008; Macůrek et al., 2008). However, different upstream kinases have been reported to mediate the activation of PLK1 homologues, suggesting that across evolution, diverse regulatory mechanisms converge to ensure PLK1 activity at mitotic entry (Zitouni et al., 2014). In human and *Xenopus*, AURORA A kinase and its cofactor BORA phosphorylate PLX1/PLK1 at Thr210/Thr201 during late G_2_ (Figure 4A) (Seki et al., 2008; Macůrek et al., 2008). Following PLK1 activation, which occurs at centrosomes at the G_2_-M transition (Bruinsma et al., 2015), PLK1 targets BORA for degradation (Chan et al., 2008). However, PLK1 localizes prominently to KTs during mitosis, thus having remained unclear how PLK1 activation status is maintained at these structures. Work in *Drosophila* and human cells suggests that AURORA B directly phosphorylates POLO/PLK1 Thr182/Thr210 T-loop at KTs (Figure 4A) (Carmena et al., 2012b; Shao et al., 2015). This is however disputed by a different study, which proposes that PLK1 activation at KTs is maintained by residual levels of AURORA A:BORA (Bruinsma et al., 2014). It is possible that both mechanisms contribute to ensure that PLK1 executes its functions as chromosomes congress to the metaphase plate (Figure 4A). An additional phosphorylation at Ser137 (Ser128 in PLX1 or Ser123 in zebrafish PLK1) has also been proposed to promote PLK1 activation at later mitotic stages (Qian et al., 1999; van de Weerdt et al., 2005). However, the downstream effect of Ser137-dependent PLK1 activation, as well as the identity of the upstream kinase, remains ill-defined.

#### KT Recruitment of PLK1

Given the large number of KT/centromere-localized substrates identified to date (Santamaria et al., 2011; Lowery et al., 2007), PLK1 is expected to be recruited to specific locations in order to execute its functions. As previously referred, many PLK1-interacting proteins are pre-phosphorylated which provides a positional cue for the appropriate localization of PLK1. These targets include proteins localized at centromeres, as well as at inner and outer KT regions, supporting the notion that PLK1 accumulates at defined pools along the centromere-KT axis (Figure 4B) (Lera et al., 2016). Remarkably, endogenous PLK1 localization was determined as a precise spot at the KT-proximal centromere or inner KT region in human RPE1 cells (Lera et al., 2016). This spatial localization coincides with that of known PLK1 binding partners, including INCENP (Carmena et al., 2012b; Goto et al., 2006) and PBIP1/CENP-U (Kang et al., 2006; Singh et al., 2021) (Figure 4B). In addition, SHUGOSHIN1 has been proposed to play a role in PLK1 recruitment, although a direct interaction between both proteins remains to be demonstrated (Pouwels et al., 2007). In turn, BUB1 (Qi et al., 2006; Singh et al., 2021), BUBR1 (Elowe et al., 2007; Suijkerbuijk et al., 2012), CLASP2 (Maia et al., 2012) and NUDC (Nishino et al., 2006) have all been proposed to contribute to the enrichment of PLK1 at the outer KT. Finally, the existence of a PLK1 pool at the outermost KT region, known as the fibrous corona, has also been hypothesized in light of recent results showing that DYNACTIN subunit p27 (Yeh et al., 2013) and CLIP-170 (Amin et al., 2014), two corona components, bind and recruit PLK1. The role of DYNACTIN in targeting PLK1 to KTs is surprising given that depletion of DYNACTIN from human cells has been shown to cause no effect on chromosome alignment, which is in contrast to the typical PLK1 depletion/inhibition phenotype (Raaijmakers et al., 2013).

#### PLK1 as a KT-MT Attachment Stabilizer

The deregulation of PLK1 activity results in a conspicuous defect in chromosome congression and segregation, arguing that PLK1 has a critical function in the regulation of KT-MT end-on attachments. However, the exact role of PLK1 has been difficult to ascertain, with an increasing amount of data supporting PLK1 involvement in promoting both the stabilization and destabilization of KT-MT interactions. Initial reports directly addressing the impact of PLK1 inactivation in spindle formation and in the interactions between chromosomes and spindle MTs in human cells revealed that PLK1 activity is required to assemble a functional bipolar spindle, which is in line with the phenotype described for *polo* mutants in *Drosophila* (Sunkel and Glover, 1988; Lénárt et al., 2007; Van Vugt et al., 2004; Sumara et al., 2004). A compromised spindle following RNAi-mediated depletion of PLK1 or inhibition of PLK1 activity with the highly potent inhibitor BI2536 likely results from loss of γ-TUBULIN and consequent reduced capacity for MT nucleation at centrosomes (Lénárt et al., 2007; Van Vugt et al., 2004; Sumara et al., 2004). Moreover, the deleterious effect that PLK1 inactivation has on spindle formation may induce a secondary outcome in the form of defective KT-MT interactions that undermine proper chromosome alignment. Nevertheless, PLK1 also appears to be directly involved in the regulation of attachment stability at the KT-MT interface, thereby ensuring efficient chromosome congression and, ultimately, faithful chromosome segregation. Although PLK1 has been proposed to modulate the stabilization of KT-MT end-on attachments through phosphorylation of different protein targets, including CLIP170 (Li et al., 2010), CLASP2 (Maia et al., 2012), Astrin (Chung et al., 2016; Geraghty et al., 2020) or SGT1 (Liu et al., 2012a), the best studied signaling pathway occurs through phosphorylation of BUBR1 (Figure 4B) (Suijkerbuijk et al., 2012; Kruse et al., 2013; Elowe et al., 2007; Matsumura et al., 2007). Mechanistically, BUBR1 is primed by CDK1 which creates a docking site for PLK1 at the outer KT. Then, PLK1 phosphorylates BUBR1 in its kinetochore attachment regulatory domain (KARD) which, in turn, recruits the PP2A:B56 phosphatase, resulting in a steady increase in phosphatase activity at KTs during prometaphase (Figure 4B) (Suijkerbuijk et al., 2012; Kruse et al., 2013; Xu et al., 2013a). The recruitment of PP2A:B56 has been proposed to counteract the activity of AURORA B during early mitosis allowing KT-MT interactions to be gradually stabilized (Figure 4B) (Foley et al., 2011). Accordingly, in the absence of PLK1 activity or in the presence of a BUBR1 KARD mutant unable to recruit PP2A:B56, stable KT-MT end-on attachments cannot be formed (Lénárt et al., 2007; Suijkerbuijk et al., 2012).

Loss of PLK1 activity does not completely abrogate the capacity of KTs to establish end on binding to MTs, but instead prevents stabilization of these interactions. This is supported by EM-based analysis revealing the presence of MTs at the surface of PLK1-depleted KTs (Van Vugt et al., 2004). However, the lack of MTs embedded in KTs in BI2536-treated cells has also been reported (Lénárt et al., 2007). Each study used different approaches to abolish PLK1 activity, which may result in distinct inactivation levels. Given PLK1 pleiotropy, it is possible that different PLK1 inactivation levels can hinder distinct PLK1 functions to different degrees and, therefore, induce alternative phenotypes (Lera and Burkard, 2012). Notably, if PLK1 is inactivated with low nanomolar concentrations of BI2536 to avoid pleiotropic defects, this is still enough to cause clear defects in both chromosome alignment and segregation (Lera and Burkard, 2012; Addis Jones et al., 2019; Lera et al., 2019). Using such an approach, recent studies propose that the KT-MT attachment defects observed upon PLK1 downregulation can result from a previously undescribed role for PLK1 in protecting centromeric integrity in response to MT-dependent pulling forces (Addis Jones et al., 2019; Lera et al., 2019). Interestingly, in BI2536-treated RPE1 cells, chromosomes were able to align at the metaphase plate and then underwent a misalignment event. Moreover, lagging chromosomes were detected during anaphase. Detailed inspection of the KT structure revealed that these misaligned or lagging chromosomes lacked many KT components, including CENP-C, CENP-T and NDC80 ^180,181^. This defect was attributed to uncontrolled DNA unwinding as a result of MT pulling forces that culminated in the disruption of centromeres. As centromere disruption alleviates tension, KT-MT end-on attachments were destabilized. In light of these results, both studies proposed a model where PLK1 activity is required to limit the access of DNA helicases like PICH and BLM, thereby securing centromere integrity in response to tension exerted due to KT-MT interactions. Consistent with the model, BI2536-dependent centromere disruption was partially rescued following PICH or BLM depletion or upon incubation with a MT-depolymerizing agent (Addis Jones et al., 2019; Lera et al., 2019). Therefore, PLK1 also contributes to the formation of KT-MT interactions by safeguarding KT/centromere structure against MT pulling forces.

#### PLK1 as a KT-MT Attachment Destabilizer

As mentioned before, PLK1 also actively promotes the stabilization of KT-MT end-on attachments by driving the recruitment of PP2A:B56 to KTs (Figure 4B). This function of PLK1, together with AURORA B activity, are likely important to support dynamic KT-MT interactions by creating cycles of MT attachment/detachment required for efficient error correction. In line with this notion, expression of a constitutively active version of PLK1 (PLK1-Thr210Asp), targeted to KTs by means of a NDC80 fusion, reduced MT turnover and compromised chromosome segregation fidelity in human HeLa cells (Liu et al., 2012b). However, it is important to note that endogenous PLK1 localizes prominently at KTs/centromeres during prometaphase and to a lesser extent at metaphase, thus suggesting that PLK1 activity is necessary mostly during prometaphase (Lénárt et al., 2007; Kang et al., 2006; Carmena et al., 2012b; Ahonen et al., 2005). Moreover, although PLK1 is localized to discrete structures along the KT-centromere axis, the bulk of PLK1 appears to be localized more internally within the KT (Lera et al., 2016). Therefore, data should be interpreted with caution as artificially targeting PLK1-Thr210Asp to NDC80 may induce non-physiological effects (Liu et al., 2012b). Indeed, expression of untargeted PLK1-Thr210Asp led to a decrease in KT-MT end-on attachment stability in human RPE1 cells (Paschal et al., 2012). Moreover, increasing PLK1 activity at metaphase by specifically preventing PP1:MYPT1-mediated dephosphorylation of its T-loop caused a PLK1-dependent attachment destabilization effect (Dumitru et al., 2017). However, the same study found that the opposite effect was observed when cells were progressing through prometaphase, which is in agreement with a PLK1 role in the BUBR1-PP2A:B56 axis (Dumitru et al., 2017). Together, these results suggest that PLK1 regulates signaling mechanisms leading both to the stabilization and destabilization of KT-MT interactions (Figure 4B). It is possible that these opposite outputs are coordinated by different PLK1 pools which are recruited to distinct sites along the KT-centromere axis. An alternative and non-mutually exclusive hypothesis is that the impact of PLK1 activity on KT-MT attachment stability is dictated by access of PLK1 to a particular substrate. The distinct activation of a PLK1 target could be achieved by the specific priming by either CDK1:CYCLIN A- or CDK1:CYCLIN B. Besides showing different degradation profiles during mitosis, both CYCLINs appear to share only a limited number of interacting substrates (Pagliuca et al., 2011).

The finding that PLK1 promotes the turnover of KT-MT end-on attachments is not surprising given that it has been implicated in the regulation of known players that have key functions in driving attachment destabilization. One of these proteins is AURORA B, whose optimal activity appears to depend on PLK1 kinase (Figure 4B). However, how exactly PLK1 contributes to AURORA B activation remains ill-defined. PLK1 was found to be required for the enrichment of CPC molecules at misaligned KTs in RPE1 cells, though a similar enrichment was not observed in HeLa cells (Salimian et al., 2011). Nevertheless, PLK1 was shown to phosphorylate SURVIVIN in HeLa cells and this was required to activate AURORA B kinase during mitosis (Chu et al., 2011). Interestingly, the specific accumulation of AURORA B at KTs that had still not congressed to the metaphase plate in RPE1 cells was interpreted as a feedback mechanism between PLK1 and AURORA B to ensure robustness to KT-MT error correction process. The discrepancy in AURORA B levels at misaligned KTs detected between the two human cell lines probably helps to explain in part why non-transformed diploid RPE1 cells, but not tumor-derived aneuploid HeLa cells, progress through several rounds of error-free mitosis (Salimian et al., 2011). Thus, by promoting AURORA B activity, PLK1 participates in the generation of unstable KT-MT end-on attachments and facilitates error correction. The requirement of PLK1 activity for AURORA B function appears to be conserved, at least in *Drosophila*, where POLO depletion was described to abolish the correct localization of CPC components at centromeres (Moutinho-Santos et al., 2012; Carmena et al., 2014). Expectedly, the percentage of erroneous syntelic KT-MT interactions was increased in POLO-depleted *Drosophila* S2 cells, suggesting that KTs maintain stable attachments with spindle MTs in the absence of POLO (Moutinho-Santos et al., 2012). These results contrast with centromere disruption and loss of attachment phenotypes observed in PLK1-inactivated human cells (Addis Jones et al., 2019; Lera et al., 2019). In that respect, it is interesting to note that a PICH homologue has not been identified in *Drosophila*, arguing that POLO inactivation probably does not lead to centromere disruption in flies*.* Nonetheless, it is still unknown whether the molecular mechanisms governing POLO/PLK1-dependent regulation of AURORA B, namely SURVIVIN phosphorylation, are conserved across evolution. Finally, PLK1 can also promote AURORA B localization in human cells via HASPIN activation, which phosphorylates pH3-Thr3 (Combes et al., 2017).

In addition to regulating AURORA B, PLK1 also contributes to the destabilization of KT-MT end-on attachments through KIF2B and MCAK. In human U2OS cells, PLK1 phosphorylates KIF2B to stimulate both its KT localization and depolymerase activity (Hood et al., 2012). Replacing endogenous KIF2B with a phospho-defective version increased the frequency of lagging chromosomes in U2OS cells, indicating that mis-attachments formed during early mitosis remained uncorrected (Hood et al., 2012). A similar chromosome segregation defect was observed in HeLa cells expressing an MCAK version harboring mutations in sites targeted by PLK1. Phosphorylation by PLK1 was shown to stimulate MT binding and depolymerizing activity of MCAK (Zhang et al., 2011; Shao et al., 2015). Interestingly, PLK1-mediated phosphorylation of MCAK is observed already in early mitosis, despite current knowledge favoring a role for MCAK specifically during metaphase (Shao et al., 2015). Although it remains unclear whether these mechanisms are conserved in other systems, PLK1 appears to have a critical role in ensuring the faithful segregation of chromosomes by facilitating the correction of inappropriate KT-MT attachments through activation of MT depolymerases.

More recently, a novel function was described for *Drosophila* POLO in fine-tuning KT-MT interactions through the regulation of the RZZc-SPINDLY-DYNEIN axis (Barbosa et al., 2020a). This is in line with previous work showing that the RZZc interacts with NDC80 to inhibit its MT-binding activity (Cheerambathur et al., 2013) (discussed in the next section). Once SPINDLY:DYNEIN engages with the RZZc and removes it from KTs, the RZZc-dependent inhibitory function is alleviated and KT-MT end-on attachments are stabilized. Hence, the RZZc was proposed to prevent the premature stabilization of end-on KT-MT interactions that could otherwise compromise error correction and mitotic fidelity. Now, we have shown that POLO-mediated phosphorylation of SPINDLY at prometaphase KTs averts untimely removal of the RZZc by DYNEIN (Barbosa et al., 2020a). This finding underscores the importance of POLO kinase in promoting dynamic KT-MT interactions early in mitosis to allow error correction and maintain genome stability.

#### Multiple Mechanisms Control PLK1 Activity Throughout Mitosis

Given that PLK1 controls KT-MT interactions through a number of different substrates and at different stages of mitosis, its activity is carefully regulated in order to ensure faithful chromosome segregation. Artificially tethering active PLK1 to KTs or preventing PLK1 T-loop dephosphorylation causes pronounced defects in the turnover of KT-MT end-on attachments (Liu et al., 2012b; Dumitru et al., 2017). PLK1 is targeted for degradation during anaphase as a result of CDH1-dependent activation of the ubiquitin ligase anaphase promoting complex/Cyclosome (APC/C) (Lindon and Pines, 2004), suggesting that alternative regulatory mechanisms must exist to balance its kinase activity during earlier mitotic stages.

**FIGURE 5:**
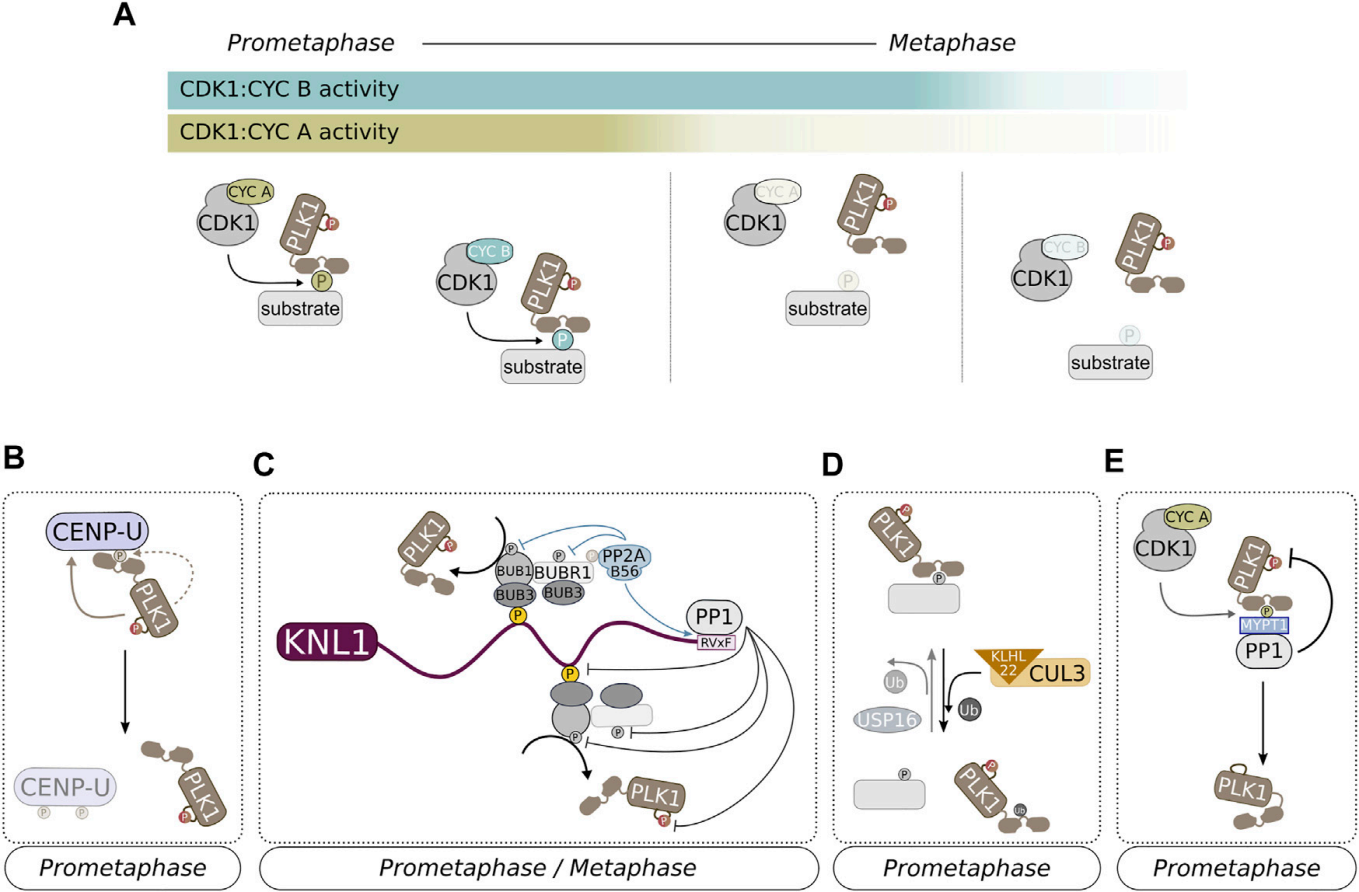
Silencing of PLK1 activity coincides with chromosomes alignment during metaphase. **(A)** The degradation of CYCLIN A and CYCLIN B at the prometaphase-to-metaphase and metaphase-to-anaphase transitions, respectively, precludes the continuous priming of PLK1 substrates and contributes to silence the activity of the kinase at KTs. **(B), (C), (D)** and **(E)** Examples of other regulatory mechanisms to control PLK1 activity and function during prometaphase and metaphase. **(B)** PLK1 promotes its own release from KTs by targeting CENP-U/PBIP1 for degradation. **(C)** PP1 bound to KNL1 has been suggested to regulate PLK1 localization during metaphase by dephosphorylating key components required for PLK1 recruitment. A similar regulatory function has been proposed for PP2A:B56, which is recruited to KTs during prometaphase. **(D)** PLK1 localization is also regulated by non-proteolytic deubiquitination/ubiquitination of the PBD. **(E)** Other regulatory mechanisms control PLK1 activation status. For instance, CDK1:CYC A primes MYPT1, a PP1 regulatory subunit, and directs it to PLK1, thereby promoting PP1-mediated dephosphorylation of PLK1’s T-loop.

Several mechanisms have been proposed to control the function of PLK1 during mitosis, promoting either the dissociation of PLK1 from KTs or a decrease in PLK1 kinase activity (Figures 5A–E). As discussed above, many PLK1 substrates are primed by CDK1:CYCLIN A and CDK1:CYCLIN B to position PLK1 close to its targets. Thus, typical degradation of CYCLIN A and CYCLIN B at the prometaphase-to-metaphase and metaphase-to-anaphase transitions, respectively, temporally regulates the access of PLK1 to its substrates (Figure 5A). Furthermore, degradation or dephosphorylation of proteins acting as PLK1 docking platforms eventually leads to loss of PLK1 recruitment and, hence, decreased local kinase activity. For instance, during prometaphase, PLK1 phosphorylates the binding partner PBIBP1/CENP-U, causing its delocalization from KTs and degradation in the cytoplasm (Figure 5B) (Kang et al., 2006; Park et al., 2015; Kang et al., 2011). Additionally, the recruitment of phosphatases to KTs also prevents excessive local PLK1 activity (Figure 5C). In fact, PLK1 levels at KTs increase upon depletion of PP2A:B56 or when PP2A:B56 fails to associate to BUBR1 during prometaphase (Figure 5C) (Foley et al., 2011; Cordeiro et al., 2020). At metaphase, the presence of PP1 appears to promote PLK1 removal from KTs (Figure 5C) (Liu et al., 2012b; Cordeiro et al., 2020). Concurrent with these mechanisms, the deubiquitination/ubiquitination cycle of PLK1 itself has been recently proposed to regulate its localization at KTs (Figure 5D) (Beck et al., 2013; Zhuo et al., 2015). During prometaphase, PLK1 accumulation at KTs requires the deubiquitinase activity of USP16 ^197^. Conversely, the non-proteolytic ubiquitylation of PLK1 seems to be sufficient to promote the release of PLK1 from KTs as human cells progress from prometaphase to metaphase (Beck et al., 2013). The E3 ubiquitin ligase CUL3-KLHL22 mono-ubiquitylates PLK1 on a key residue localized within the PBD, likely preventing the molecular interactions between the PBD and phospho-targets. Consistently, a non-ubiquitylatable version of PLK1 was found to co-immunoprecipitate with increased amounts of known PLK1 binding partners at KTs (Beck et al., 2013). Together, these mechanisms help to explain how PLK1 accumulates at KTs preferentially during prometaphase and demonstrate that PLK1 KT levels are actively regulated in human cells (Ahonen et al., 2005; Kang et al., 2006; Lénárt et al., 2007).

It is important to note that modest levels of PLK1 are still detected at aligned KTs in human cells. Moreover, in other organisms like *Drosophila* the decline in the levels of KT-localized POLO as mitosis progresses is not as evident as in human cells (Carmena et al., 2012b). Therefore, additional regulatory mechanisms must control the activity of POLO/PLK1 that remains at KTs. Dephosphorylation of Thr210 by mitotic phosphatases likely contributes to silence the activity of PLK1. As previously mentioned, PP1:MYPT1 was shown to regulate the phosphorylation status of PLK1 T-loop. Depletion of PP1:MYPT1 or its exclusion from KTs prevented Thr210 dephosphorylation in human mitotic cells (Dumitru et al., 2017; Liu et al., 2012b; Yamashiro et al., 2008; Kachaner et al., 2012). Mechanistically, CDK1:CYCLIN A phosphorylates the regulatory subunit MYPT1 and creates a docking site for PLK1 which facilitates PP1-mediated dephosphorylation of Thr210 (Figure 5E) (Dumitru et al., 2017; Yamashiro et al., 2008). In vertebrates, an additional player, OPTINEURIN, appears to stimulate the CDK1-dependent priming phosphorylation of MYPT1 (Kachaner et al., 2012). Furthermore, the T-loop of PLK1 can be dephosphorylated by KNL1-associated PP1 (Figure 5C) (Liu et al., 2012b). As PP1 levels peak at KTs during metaphase, dephosphorylation of PLK1 T-loop may correspond to a late event in the PLK1 silencing pathway. Interestingly, PLK1 was also recently shown to be post-translationally modified by SET7/9 methyltransferase (Yu et al., 2020). This enzyme catalyzes the addition of a di-methyl group to a Lys residue within the kinase domain of PLK1, effectively suppressing its kinase activity. Remarkably, a non-methylatable version of PLK1 displays high kinase activity against a KT-localized FRET sensor, similar to the kinase activity measured for the constitutively active version PLK1-Thr210Asp (Yu et al., 2020). Thus, the kinase domain of PLK1 is targeted by additional mechanisms other than those promoting the dephosphorylation of its T-loop, thereby ensuring that PLK1 activity is efficiently regulated in time and space.

### RZZc-Dependent Mechanisms

Cumulative evidence supports a conserved role for the RZZc in KT-MT attachment regulation, though the mechanistic details underlying this specific function remain elusive. While *in vitro* data shows that ROD interacts with the NDC80 tail and inhibits the affinity of the NDC80c towards the MT lattice, most *in vivo* data is based on solid, yet indirect, results showing that chromosome alignment defects occur when RZZc levels at KTs are experimentally increased (Gassmann et al., 2008; Barisic et al., 2010; Cheerambathur et al., 2013; Amin et al., 2018; Barbosa et al., 2020a). Given that the RZZc has a role in promoting the expansion of the fibrous corona, which simultaneously facilitates KT capture of spindle MTs and provides an additional platform for SAC signaling (Kops and Gassmann, 2020), a more detailed molecular characterization of RZZc activity towards the destabilization of KT-MT end on attachments is required to fully understand how this protein complex executes its mitotic functions.

#### KT Recruitment of the RZZc

**FIGURE 6:**
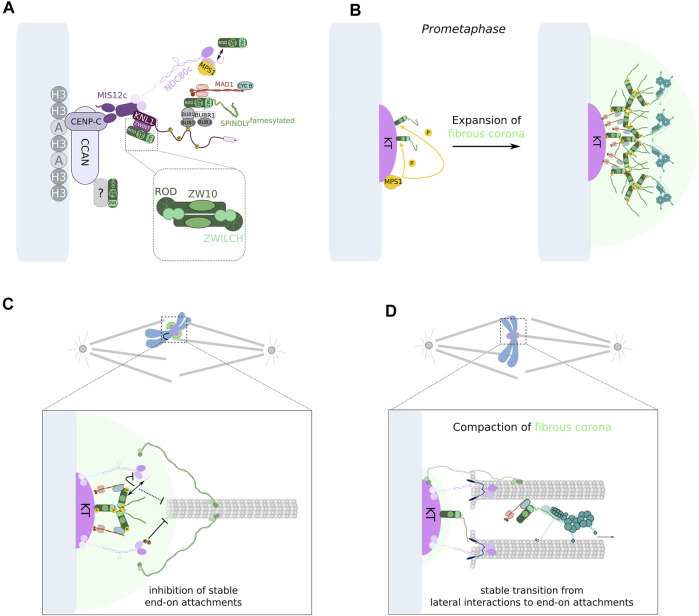
RZZc:SPINDLY-dependent regulation of KT size dynamics and KT-MT attachment stability. **(A)** Localization of the RZZc to the outer KT requires different binding partners, including ZWINT and BUB1. Unlike RZZc:ZWINT, a direct interaction between the RZZc and BUB1 has not been demonstrated yet. Moreover, additional binding partners for the RZZc are likely to exist, but still remain unidentified (“?”). It has been proposed that NDC80 may constitute an RZZ binding partner, as ROD was found to interact with the N-terminal tail in NDC80 in a yeast two hybrid assay. Furthermore, the RZZc directly recruits SPINDLY to KTs. In human cells, this interaction requires SPINDLY to be farnesylated at its C-terminal CAAX motif (SPINDLY^farnesylated^). **(B)** Expansion of the fibrous corona during prometaphase is driven by the RZZc:SPINDLY^farnesylated^ and is stimulated by MPS1-dependent phosphorylation of ROD. Importantly, SPINDLY also contributes to the subsequent compaction of the fibrous corona by recruiting DYNEIN:DYNACTIN. **(C)** The localization of different MAPs, including DYNEIN and CENP-E, to the fibrous corona facilitates initial MT capture. However, the presence of an expanded corona can inhibit the formation of stable load-bearing end-on attachments. The decreased stability of end-on attachments may result from the inhibitory action of the RZZc over NDC80/HEC1. **(D)** Compaction of the fibrous corona enables the conversion of lateral contacts between KTs and MTs into stable end-on attachments. DYNEIN:DYNACTIN has an critical function in efficiently stripping the RZZc away from KTs, thereby contributing to relieve its inhibitory activity towards KT-MT end-on attachments. DYNEIN:DYNACTIN-dependent disassembly of the fibrous corona also leads to the removal of SAC proteins and silencing of the mitotic checkpoint.

The RZZc is composed of three subunits, ROD, ZW10 and ZWILCH, whose encoding genes were originally identified in *Drosophila* (Figure 6A). Null mutations in both *rough deal* (encodes ROD) and *zeste-white 10* (encodes ZW10) were shown to invariably cause high levels of aneuploidy due to strong defects in chromosome segregation (Karess and Glover, 1989; Williams et al., 1992). Consistent with a key role in mitosis, the RZZc is well conserved among higher eukaryotes (Karess, 2005). Recently, analysis of purified RZZc enabled a comprehensive characterization of its structure. Visualization of negatively-stained RZZc by EM showed that isolated complexes appeared as a ∼42 nm long particles. Moreover, cryo-EM reconstructions of single RZZc revealed a 2-fold symmetry which is in agreement with previous molecular mass quantifications suggesting that a single RZZc assumed a 2:2:2 stoichiometry (Figure 6A) (Scaërou et al., 2001; Mosalaganti et al., 2017). Within the complex, the two ROD polypeptide chains are oriented in an anti-parallel fashion, with ZWILCH interacting both with the N- and C-termini of ROD. ZW10 binds to the central region of ROD and does not appear to establish contacts with ZWILCH (Çivril et al., 2010; Mosalaganti et al., 2017).

Despite recent advances in the characterization of the structural organization of the RZZc, the molecular factors that dictate RZZc recruitment to KTs during early mitosis remain less understood. Following the identification of human ZW10 and ROD, a yeast-two hybrid screen uncovered ZWINT as a ZW10-interacting protein (Starr et al., 2000). ZWINT localizes at KTs through an interaction between its N-terminal domain and the C-terminal coiled coil domain of KNL1 (Vos et al., 2011; Petrovic et al., 2010). ZWINT localization precedes ZW10 recruitment, thus suggesting that ZWINT could serve as a docking site for the RZZc at KTs (Figure 6A) (Starr et al., 2000). In agreement with this hypothesis, depletion of ZWINT was shown to prevent the accumulation of RZZc at KTs (Wang et al., 2004; Kops et al., 2005). Importantly, AURORA B was proposed to regulate the interaction between ZWINT and the RZZc (Kasuboski et al., 2011). However, this was not consistently reported in other studies that, instead, failed to detect a similar defect in the recruitment of RZZc or RZZc-binding partners following AURORA B inhibition (Barisic et al., 2010; Famulski and Chan, 2007; Matson and Stukenberg, 2014; Ying, 2009; Famulski et al., 2008). Moreover, ZWINT does not appear to be conserved in *Drosophila* (Karess, 2005) and is dispensable for RZZc recruitment to KTs in *C. elegans* (Varma et al., 2013), hinting for the existence of additional factors that contribute to the KT localization of the complex. One such recruiting factor could be BUB1, in particular, its central domain, although more recent data argues against a stringent requirement for BUB1 in RZZc recruitment (Figure 6A) (Caldas et al., 2015; Silió et al., 2015; Zhang et al., 2015; Qian et al., 2017; Rodriguez-Rodriguez et al., 2018). Together with a lack of evidence for a direct interaction between BUB1 and any of the RZZc subunits, the results described so far indicate that, in addition to BUB1 and ZWINT, other contacts between the KT and the RZZc remain to be identified. One candidate is the NDC80c, which has been shown to interact with ROD *in vitro* in *C. elegans* (Cheerambathur et al., 2013). In *Drosophila* S2 cells, where the RZZc is also recruited to KTs in a KNL1-and BUB1-independent manner, ZW10 was demonstrated to bind directly to CAL1, a fly-specific CID/CENP-A chaperone that localizes to the inner KT (Pauleau et al., 2019).

#### The Role of the RZZc in KT-MT Interactions

At KTs, the RZZc has critical functions in SAC signaling and, as more recently proposed, in fine-tuning KT-MT end-on attachments (Kops and Gassmann, 2020; Barbosa et al., 2020b). Depletion of the RZZc in HeLa and *Drosophila* cells results in a clear reduction of MAD1:MAD2 at KTs, suggesting that the RZZc operates in SAC signaling by providing a KT platform for MAD1:MAD2 (Figure 6A) (Kops et al., 2005; Buffin et al., 2005). However, the precise requirement for the RZZc-dependent localization of MAD1:MAD2 remains controversial (Kops and Gassmann, 2020). In addition to a role in the recruitment of MAD proteins, the RZZc regulates SAC signaling at KTs by targeting DYNEIN:DYNACTIN (Figures 1A,B protein complex implicated in the removal of SAC proteins from KTs (Wojcik et al., 2001; Howell et al., 2001). DYNEIN has multiple binding partners that support its localization to diverse intracellular sites and likely help to activate DYNEIN motor function at these locations. The RZZc itself has a key role in directing the recruitment of DYNEIN to KTs via a direct interaction between ZW10 and DIC (Whyte et al., 2008). Moreover, ZW10 was shown to interact with DYNAMITIN/p50, a subunit of the DYNACTIN complex, thereby facilitating the enrichment of DYNEIN:DYNACTIN at KTs (Starr et al., 1998). The RZZc further recruits SPINDLY, a DYNEIN adaptor present at KTs that is important for the assembly of a functional DYNEIN complex at this location (Figure 1B) (Griffis et al., 2007; Gassmann et al., 2010). Therefore, the RZZc is involved in coordinating the maintenance and silencing of the SAC response. This is achieved by enabling the KT recruitment of SAC effectors, as well as of the molecular machinery that transports these effector proteins away from the signaling hub. Importantly, the RZZc-dependent recruitment of DYNEIN:DYNACTIN is critical for timely compaction of the fibrous corona, a process that is intimately linked to the establishment of appropriate KT-MT end-on attachments.

By the time the mitotic spindle starts to assemble, the fibrous corona changes its shape and volume to ultimately increase its size by several nm, thus accelerating KT capture of MTs and re-orientation and possibly increasing SAC robustness (Figure 6B) (Kops and Gassmann, 2020; Barbosa et al., 2020b). Not surprisingly, the fibrous corona accumulates numerous KT proteins, including SAC-related proteins such as the RZZc, SPINDLY, MAD1:MAD2, CYCLIN B, as well as MT attachment-related MAPs like DYNEIN:DYNACTIN, CENP-E and CENP-F (Figure 1B). Among these, the RZZc and SPINDLY, which are known to interact with each other, have crucial roles in driving the expansion of the fibrous corona in human cells (Figure 6B) (Kops and Gassmann, 2020; Barbosa et al., 2020b; Raisch et al., 2021). As mentioned before, SPINDLY is also critically involved in the compaction of the fibrous corona by supporting the localization of DYNEIN:DYNACTIN which actively removes corona components. In addition to the aforementioned role in facilitating SAC silencing, the DYNEIN-mediated stripping of corona proteins also contributes to the formation of stable end-on KT-MT interactions. In fact, the corona expansion occurs only transiently and its disassembly constitutes a decisive step in the KT-MT end-on attachment process, as it is required to allow the stable transition from a lateral interaction mode to an end-on attachment configuration (Figures 6C,D) (Kops and Gassmann, 2020; Barbosa et al., 2020b). The inability of expanded KTs to establish end-on interactions with MTs probably stems from the recently proposed RZZc-mediated inhibition of NDC80c (Cheerambathur et al., 2013). To substantiate this model, yeast-two hybrid assays revealed that the ROD β-propeller domain interacts with the NDC80 N-terminal tail. Moreover, *in vitro* experiments with reconstituted complexes from *C. elegans* demonstrated that the NDC80c was unable to interact with MTs when a minimal RZZc comprising the ROD-1 β-propeller and ZWL-1/ZWILCH was present. Given that in *C. elegans*, the NDC80 tail is not required for robust KT-MT end-on attachments *in vivo*, the interaction with the RZZc was then proposed to negatively affect the MT-binding activity of the adjacent CH domain in NDC80 (Figure 6C) (Cheerambathur et al., 2013).

Although a similar interaction between RZZc and NDC80c has not been confirmed in other organisms, several observations argue in favor of a conserved role for the RZZc in the regulation of NDC80c function and, thus, of KT-MT attachments. Most studies rely on the phenotypic analysis of chromosome congression in a context where RZZc cannot be removed from KTs, a condition that can be achieved through the depletion of SPINDLY or DYNEIN (Gassmann et al., 2008; Barisic et al., 2010; Amin et al., 2018; Barbosa et al., 2020a). SPINDLY was originally identified in two independent RNAi-based screens for proteins required for mitotic progression and cell morphology in *Drosophila* and its depletion resulted in a delayed progression accompanied by chromosome alignment defects (Griffis et al., 2007). This phenotype was consistently observed across different model organisms and was significantly rescued when the RZZc was co-depleted with SPINDLY (Gassmann et al., 2008; Barisic et al., 2010; Gassmann et al., 2010; Amin et al., 2018; Barbosa et al., 2020a). Moreover, when the RZZc persists at KTs in *Drosophila* cells, by depleting either SPINDLY or DYNEIN, KTs take longer to progress from a lateral interaction to an end-on KT-MT attachment (Barbosa et al., 2020a). In agreement with this, in human cells with persistent expanded coronas, both end-on and lateral interactions can occur coincidently in the same KT, thus suggesting that the RZZc inhibits the establishment of mature end-on KT-MT interactions (Sacristan et al., 2018). Such an RZZc-dependent mechanism may be important to prevent the premature stabilization of erroneous attachments during early mitosis. An increase in the number of incorrect KT-MT attachments could then overload the error correction machinery and, thus, compromise mitotic fidelity (Cheerambathur et al., 2013).

Importantly, transient end-on interactions may occur even in the presence of increased levels of RZZc, possibly through the activity of MAPs other than the NDC80c (Sacristan et al., 2018). In *Drosophila* and human cells, the RZZc is not completely removed from KTs by metaphase, suggesting that RZZc levels below a certain threshold do not inhibit the MT-binding activity of NDC80c (Karess, 2005; Gassmann et al., 2010). Therefore, a transient end-on attachment configuration would allow DYNEIN-mediated removal of the RZZc and a consequent relief of NDC80c inhibition, further promoting the formation of stable KT-MT interactions (Figure 6D). Interestingly, low MT occupancy was proposed to trigger a significant loss of DYNEIN cargos like MAD1 and ZW10 from KTs (Etemad et al., 20192019; Kuhn and Dumont, 2019). The rate of removal was however slower in these low MT-attached KTs (Kuhn and Dumont, 2019). Blocking the rapid loss of the RZZc from KTs could in principle contribute to a robust fine-tuning of KT-MT end-on attachments by enabling the RZZc-dependent regulatory mechanism outlined above. It has been recently shown that POLO/PLK1 may have an important role in restricting the release of the RZZc from KTs, thereby promoting appropriate turnover of KT-MT end-on attachments (Barbosa et al., 2020a). Importantly, this may constitute a regulatory pathway controlling the activity of a specific DYNEIN module (RZZc:SPINDLY:DYNEIN). CENP-F has also been suggested to restrict DYNEIN-mediated removal of corona components in a NDE1-dependent manner. However, a CENP-F:NDE1-dependent mechanism appears to regulate the release of several DYNEIN cargos, indicating that it broadly regulates DYNEIN function in fibrous corona assembly dynamics (Auckland et al., 2020). Curiously, preventing CENP-F:NDE1 from blocking excessive removal of corona components, including the RZZc, caused an increase in chromosome segregation errors in human HeLa cells. Despite the rapid loss of the RZZc—and the fibrous corona –, these cells had only milder defects in chromosome congression, arguing that chromosome capture was not severely impaired (Auckland et al., 2020). Therefore, the observed increased rate of chromosome mis-segregation could potentially result from a deficient RZZc-mediated regulation of KT-MT attachment fidelity.

## Concluding Remarks

The correct segregation of chromosomes at the end of mitosis relies on the establishment of appropriate attachments between KTs and MTs. The formation of amphitelic interactions, whereby sister KTs bind to MTs extending from opposite spindle poles, ensures that chromatids are equally distributed among the newly arising daughter cells. However, KTs and MTs interact stochastically during early mitotic stages and erroneous attachment configurations (syntelic and merotelic) can be established. This review highlights some of the main molecular mechanisms that prevent and resolve aberrant KT-MT interactions. Key mitotic kinases, such as AURORA B and PLK1, invariably participate in the regulation of KT-MT end-on attachment stability by targeting multiple effector proteins at the KT-MT interface.

AURORA B ensures reversible end-on attachments by phosphorylating NDC80 which represents the major KT-MT coupling element. Furthermore, AURORA B negatively affects additional MAPs that otherwise contribute to strengthen the interactions between KTs and the plus ends of MTs. Given that AURORA B is found at distinct sites across the KT-centromere axis, different models have been put forward to explain how the kinase activity is regulated during mitosis. The prevailing models describe that tension established across sister KTs as a result of correct bioriented attachments selectively suppresses AURORA B function towards KT substrates, further stabilizing KT-MT interactions. These models support the observation that the centromeric pool of AURORA B is critically required to dissolve mal-oriented attachments. However, such models have to be reconciled with the current view that a pool of AURORA B also localizes at KTs. This demands additional efforts to understand how the function of KT AURORA B is regulated to ultimately allow the stabilization of KT-MT end-on attachments. Moreover, it is still unknown whether centromeric- and KT-localized AURORA B phosphorylate different substrates. Because AURORA B localization changes with the establishment of inter-KT tension and its activity can lead either to MT detachment or MT depolymerization from KTs, it will be interesting to address whether each outcome is a consequence of specific substrate phosphorylation.

Although a comprehensive overview is still missing, the results reported thus far suggest that PLK1 functions to keep KT-MT attachment stability under an optimal dynamic range that not only allows MT capture, but also facilitates error correction. It is noteworthy that PLK1 activity decreases as cells reach metaphase, which coincides with the gradual increase in the stability of KT-MT end-on attachments. It is possible that PLK1 controls highly responsive pathways that integrate both functions and future work will be required to fully understand how PLK1 activity is stringently regulated to elicit opposite actions towards KT-MT interactions. An interesting possibility is that, as PLK1 substrates require a priming phosphorylation driven by CDK1, specificity in the molecular signaling outcome could be provided by the different CDK1-associated CYCLINs.

AURORA B has been regarded as the main regulator of the MT-binding activity of the NDC80c. However, recent studies unraveled a role for the RZZc in the destabilization of NDC80c-mediated KT-MT end-on attachments. The RZZc localizes prominently at KTs during early mitosis and drives the expansion of the fibrous corona, which is expected to accelerate MT capture. However, the presence of RZZc is also likely to be important to inhibit premature KT-MT end-on interactions that may form inappropriately, such as merotelic attachments. Although an *in vitro* interaction between ROD and NDC80 has already been described, further validation and characterization of this interaction will be crucial to fully understand how the RZZc inhibits the establishment of KT-MT end-on attachments.

Importantly, the upstream regulation of KT-MT attachments by AURORA B and PLK1 must be coordinated with the opposing activities of mitotic phosphatases. This dynamic cycle of phosphorylation/dephosphorylation ensures the fine-tuning of KT-MT interactions in a highly responsive manner so that the turnover of KT-MT attachments coupled with the selective stabilization of correct interactions is able to support the faithful segregation of chromosomes.
